# Host neuronal PRSS3 interacts with enterovirus A71 3A protein and its role in viral replication

**DOI:** 10.1038/s41598-022-17272-2

**Published:** 2022-07-27

**Authors:** Patthaya Rattanakomol, Potjanee Srimanote, Pongsri Tongtawe, Onruedee Khantisitthiporn, Oratai Supasorn, Jeeraphong Thanongsaksrikul

**Affiliations:** 1 Graduate Program in Biomedical Sciences, Faculty of Allied Health Sciences, Thammasat University, Pathum Thani, 12120 Thailand; 2 Thammasat University Research Unit in Molecular Pathogenesis and Immunology of Infectious Diseases, Thammasat University, Pathum Thani, 12120 Thailand; 3 Department of Medical Technology, Faculty of Allied Health Sciences, Thammasat University, Pathum Thani, 12120 Thailand

**Keywords:** Microbiology, Virology, Virus-host interactions

## Abstract

Enterovirus A71 (EV-A71) causes hand, foot, and mouth disease associated with neurological complications in young children. Currently, there is no specific treatment for EV-A71 infection due to the inadequate information on viral biology and neuropathogenesis. Among enteroviruses, nonstructural 3A protein mediates the formation of replication organelles which plays a major role in viral RNA synthesis and assembly. Although enteroviral 3A proteins have been intensively studied, the data on EV-A71 3A, especially in neuronal cells, are still limited. In this study, PRSS3 (mesotrypsinogen, also known as brain trypsinogen) was identified as EV-A71 3A-interacting counterpart from the transfected human neuroblastoma SH-SY5Y cells by pull-down assay and liquid chromatography tandem mass spectrometry. It was confirmed that PRSS3 variant 3 derived from human SH-SY5Y cells had the physical interaction with EV-A71 3A. Importantly, the role of PRSS3 in EV-A71 replication was verified by overexpression and siRNA-mediated gene silencing approaches. The detailed mechanism of the PRSS3 involved in EV-A71 replication and neuropathogenesis warrants further experimental elucidation. In conclusion, this study has discovered a novel EV-A71 3A interacting protein that offers the opportunity to study the neuropathogenesis of the infection which paves the way for developing a specific and effective treatment for the disease.

## Introduction

Hand, foot, and mouth disease (HFMD) is an illness mostly found in children at the age under 5 years caused by the viruses classified in the family *Picornaviridae*. HFMD has become a public health problem in Asia–Pacific countries including China, Hong Kong, Taiwan, Japan, Malaysia, Singapore, Vietnam, Cambodia, and Thailand^1–9^. The Asia–Pacific region has experienced the cyclical large outbreaks of HFMD that occur every 1–2 years and potentially become an endemic viral infection^10^. Enterovirus A71 (EV-A71) is one of the main causative agents of HFMD. The symptoms range from mild or typical HFMD to severe HFMD. The typical HFMD symptoms are blisters or sores in the mouth and rash on the hands, feet, and buttocks as well as legs which they are self-limited within seven to ten days after the symptom onset. While the severe HFMD involves the ability of EV-A71 to invade the central nervous system (CNS) resulting in neurological complications such as meningitis, brainstem encephalitis, acute flaccid paralysis, neurogenic cardio-respiratory failure and even death. The neurological complications of the CNS are consequences of the viral replication and inflammation of viral-infected tissues^11^. Pieces of evidence from in vitro and animal experiments have suggested that enteroviruses gain an access to the CNS by three main pathways which including direct infection of brain microvascular endothelium, Trojan horse invasion through infected leukocytes, and retrograde axonal transport^12^. However, the pathogenesis mechanism of the EV-A71 infection in the CNS has not been elucidated yet, resulting in a lack of specific antiviral drugs for treatment. Therefore, the vaccine has been highlighted as a major role in controlling the disease. Three inactivated monovalent EV-A71 vaccines were approved for exclusively used in China^12^. These vaccines were produced from C4 subgenotype EV-A71, which did not confer protection against infection by different genotypes of EV-A71.

The positive-sense RNA genome of EV-A71 contains a single open reading frame (ORF) encoding for a polyprotein that is subsequently processed into structural proteins (VP1-VP4) and nonstructural proteins including 2A, 2B, 2BC, 2C, 3A, 3AB, 3B, 3C, 3CD, and 3D which play diverse roles in the viral replication^12–15^. To establish a successful infection, the virus has to produce many viral progenies throughout the duration of the infection. Moreover, at the same time, the virus has to evade or protect itself from pre-mature termination of its replication by the host intracellular immune recognition. To evade the host intracellular immune sensors, the replicative double-stranded RNA molecules of enterovirus generated during genome replication are hidden within virally-induced vesicles termed as replication organelles (ROs)^16,17^. The remodeling of the host intracellular membrane to generate the ROs is mediated by the orchestrated actions of both viral nonstructural proteins (i. e. 2C, 3A, 3B, 3AB, 3CD, and 3D) and host cellular proteins and lipids^16,17^. Utilization of the host proteins and lipids for ROs formation is diverse and varies among the enteroviruses, depending on the host cells and the preferential location of ROs formation, such as endoplasmic reticulum (ER), Golgi apparatus, peroxisomes, endosomes, mitochondria, or plasma membrane^16,17^. In many picornaviruses including EV-A71, 3A proteins, which highly conserved among enteroviruses, have been reported to interact with the acyl-CoA binding domain containing 3 (ACBD3) to recruit PI4KIIIβ to the membrane^18–20^. PI4KIIIβ functions in the generation of phosphatidylinositol-4-phosphate (PI4P) by phosphorylation of phosphatidylinositol (PI). Picornaviruses generate the ROs that are enriched with PI4P for providing the interaction with viral RNA-dependent RNA polymerase (RdRp) 3D protein^21,22^. Cellular proteins containing the PI4P-binding domain, called the pleckstrin-homology domain (PH), such as oxysterol-binding protein (OSBP), ceramide transfer protein (CERT), and four-phosphate-adaptor protein 1 and 2 (FAPP1/FAPP2), can specifically sense and bind to PI4P lipids^23,24^. The OSBP has been reported to be recruited to the ROs and plays a key role in the transport of cholesterol and PI4P between the ER and Golgi^25^. Although the roles and the functions of enteroviral 3A proteins have been intensively studied, the data on the biology and cellular function of EV-A71 3A protein, in particular in neuronal cells, are still limited.

In this study, the interacting counterpart of the EV-A71 3A protein was identified in human neuroblastoma SH-SY5Y cells and its role in the viral replication was investigated. Since the viral nonstructural protein might contribute to the viral pathogenesis, the gained knowledge from our research would provide a better understanding on the pathogenesis mechanism of the EV-A71 infection associated with severe neurological complications. The more interacting counterparts of the EV-A71 3A protein discovered the more 3A interactome expanded, which could be further studied for the identification and development of a biomarker for prognosis prediction and drug targets. The methodology and results were mentioned herein.

## Results

### Identification of EV-A71 3A interacting protein in human neuronal SH-SY5Y cells by immunoprecipitation assay and liquid chromatography tandem mass spectrometry (LC–MS/MS)

**Figure 1:**
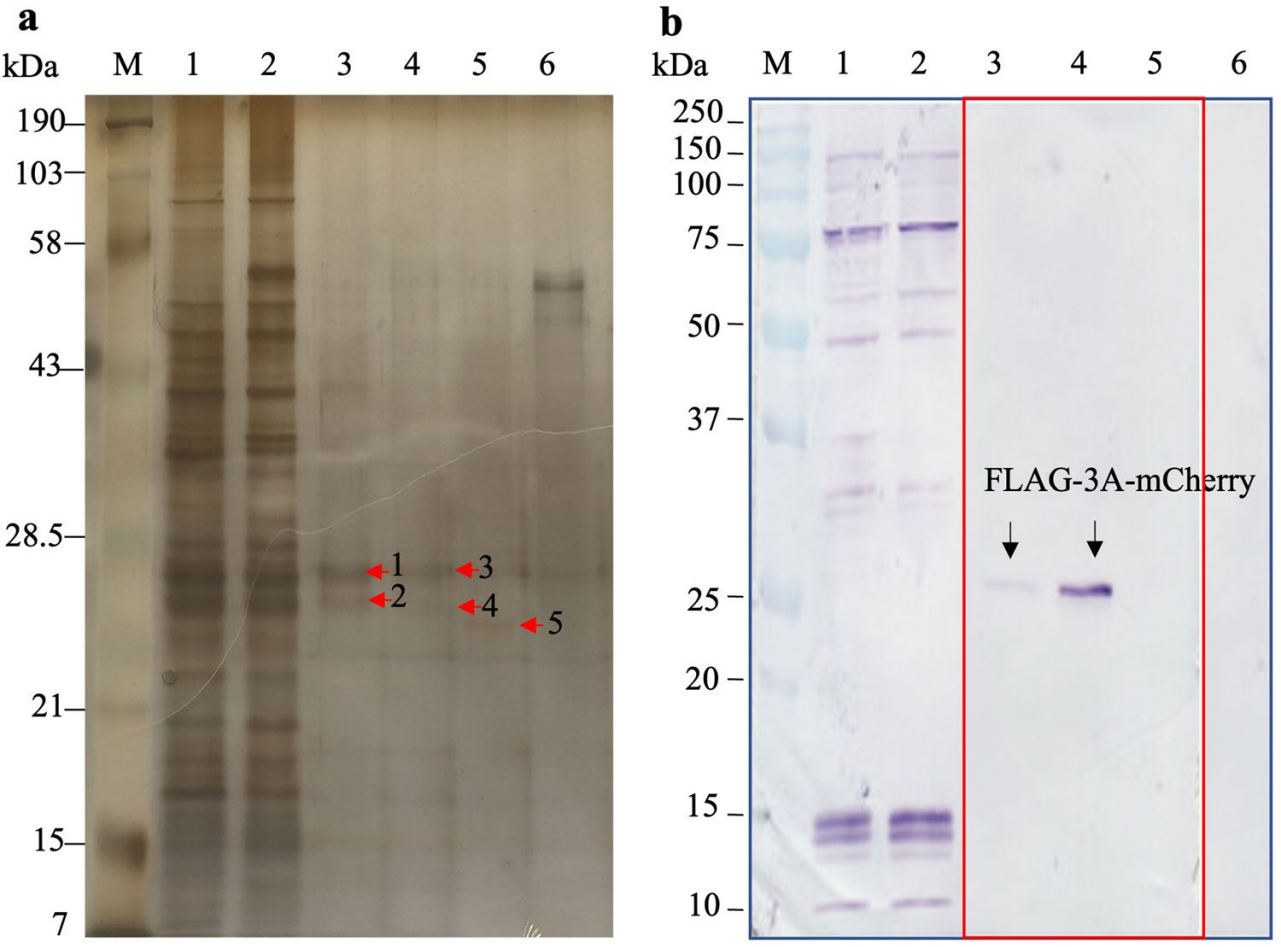
(**a**) Pull down of proteins with EV-A71 3A in the cell lysate prepared from *pLVX-Puro::FLAG-3A-mCherry*-transfected SH-SY5Y cells. Lane 1, cell lysate prepared from untransfected SH-SY5Y cells; lane 2, cell lysate prepared from *pLV-mCherry* transfected SH-SY5Y cells; lanes 3–5, eluate fractions from pull down by anti-FLAG M2 magnetic beads; lane 6, last washed fraction. The proteins were resolved in 12% SDS-PAGE and stained with Coomassie brilliant blue followed by counterstaining with silver dye. Red arrows indicate distinguishable protein bands excised and subjected for protein identification. (**b**) Western blot analysis of the respective pull down fractions of (**a**) to detect the FLAG-3A-mCherry (indicated by arrows) using rabbit anti-mCherry polyclonal antibody. The lanes 3–5 (in red box) on blotted membrane were cropped and moved to area on the membrane that align lanes with those in the gel (**a**). The original gel of Fig. 1a was shown in Supplementary Fig. 2.

**Figure 2:**
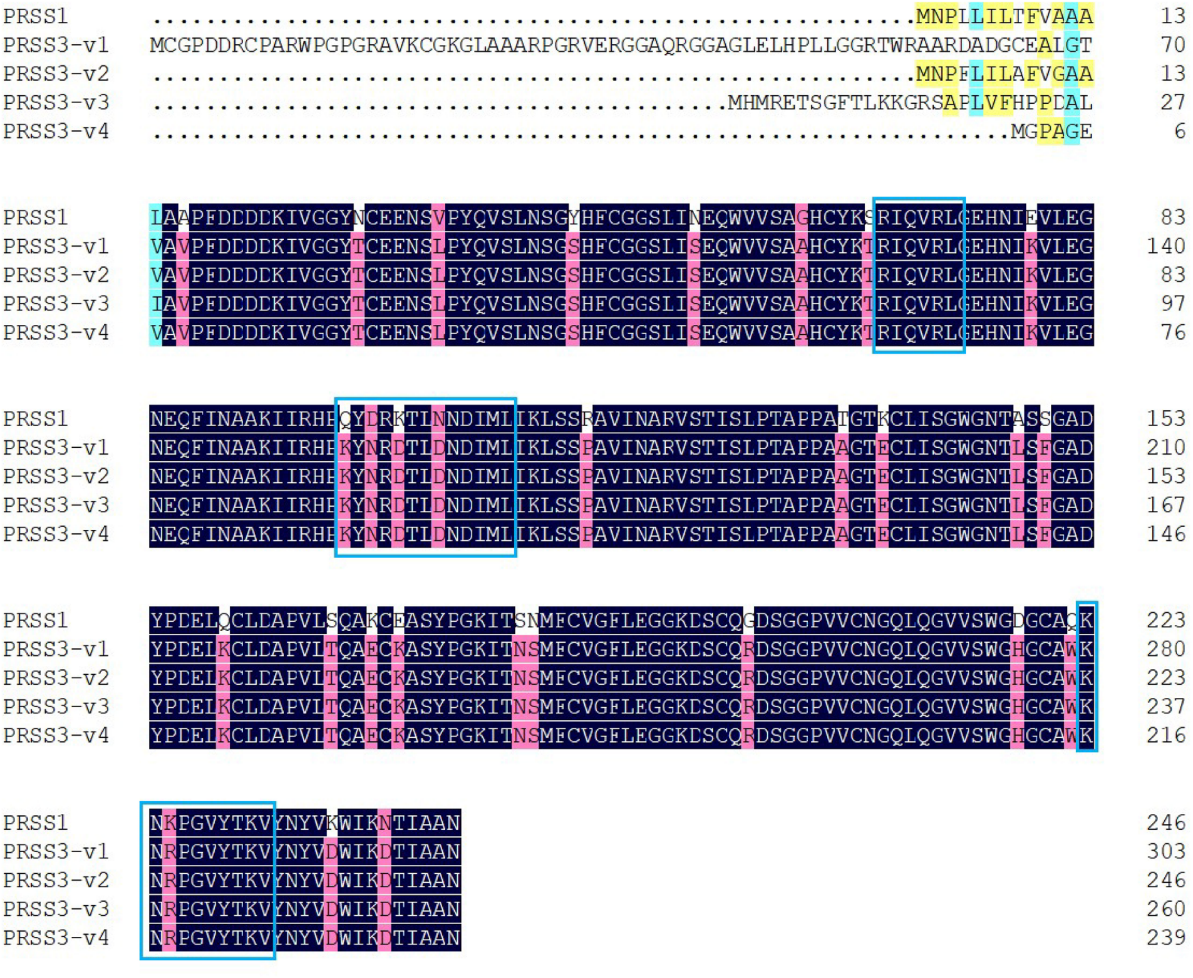
Multialignment of amino acid sequences of PRSS1 and four PRSS3 transcript variants (PRSS3 variants 1–4; PRSS3-v1 to PRSS3-v4 ) by DNAMAN program. Highlight homology level: blue = 100%, pink ≥ 75%, cyan ≥ 50%, and yellow ≥ 33%. The peptide sequences generated by LC-MS/MS including RIQVRL, KTLNNDIMLIKL, and KNKPGVYTKV, identified as PRSS1 or PRSS3 were highly similar to amino acid sequences shown in the blue boxes.

To investigate the EV-A71 3A interacting proteins in the human neuronal cells, a recombinant *pLVX-Puro::FLAG-3A-mCherry* plasmid expressing the EV-A71 3A protein fused with FLAG-tag and mCherry protein, namely, FLAG-3A-mCherry, was constructed and used to transfect the human neuroblastoma SH-SY5Y cells. The cells transfected with *pLVX::FLAG-mCherry* served as pull-down background control. The eluates from the FLAG pull-down of the SH-SY5Y transfected cells were resolved by SDS-PAGE and detected by Coomassie brilliant blue followed by counterstaining with silver dye. The distinguishable protein bands present in the eluates of the FLAG-3A-mCherry-transfected cells were compared with the protein bands present in the eluates of the pull-down background control and the protein background controls (Fig. 1a). The expression of the FLAG-3A-mCherry and achievement of pull-down was validated by Western blot analysis (Fig. 1b). The SDS-PAGE and Western blot analysis of eluates from FLAG pull-down of the FLAG-mCherry-transfected cells were provided as Supplementary Fig. 1. Five distinguishable bands were selected, excised, and subjected to trypsin digestion and protein identification by LC-MS/MS. By using Gene Tools Program, the predicted relative molecular weights of the selected protein bands were approximately 22–26 kDa. Analysis of the mass spectral data using Mascot software identified potential EV-A71 3A interacting proteins with 95% confidence (Table 1). PRSS1 (cationic trypsinogen), PRSS3 (mesotrypsinogen), putative protein N-methyltransferase FAM86B1, and proteasome subunit alpha type-7 were predicted from the selected protein bands. PRSS3 (mesotrypsinogen) and PRSS1 (cationic trypsinogen) were the proteins of interest because their Mascot scores were higher than those of the other identified proteins. Their molecular weights were also close to the predicted molecular weights of the selected protein bands resolved in SDS- PAGE gel (approximately 22–26 kDa). The human trypsinogen PRSS1 and PRSS3 showed a high sequence homology as analyzed by the sequence alignment (Fig. 2). Therefore, the protein identification by LC-MS/MS generated peptide sequences could be interpreted as PRSS1 or PRSS3.

**Table 1:** List of the proteins identified by LC-MS/MS.

Identified protein	MW (kDa)	Accession number	Band number	Mascot score
PRSS3 protein [*Homo sapiens*]	26.5	A1A508	1	63
			2	77
			3	65
			4	57
			5	51
Cationic trypsinogen, partial [*Homo sapiens*]	9.1	AAG30948.1	1	61
			2	78
			3	68
			4	60
			5	51
Proteasome subunit alpha type-7 [*Homo sapiens*]	27.9	O14818	1	48
Putative protein N-methyltransferase FAM86B1 [*Homo sapiens*]	48.3	R4GN25	2	49

### Determination of PRSS expression in SH-SY5Y cells by RT-PCR and immunofluorescence assay

**Figure 3:**
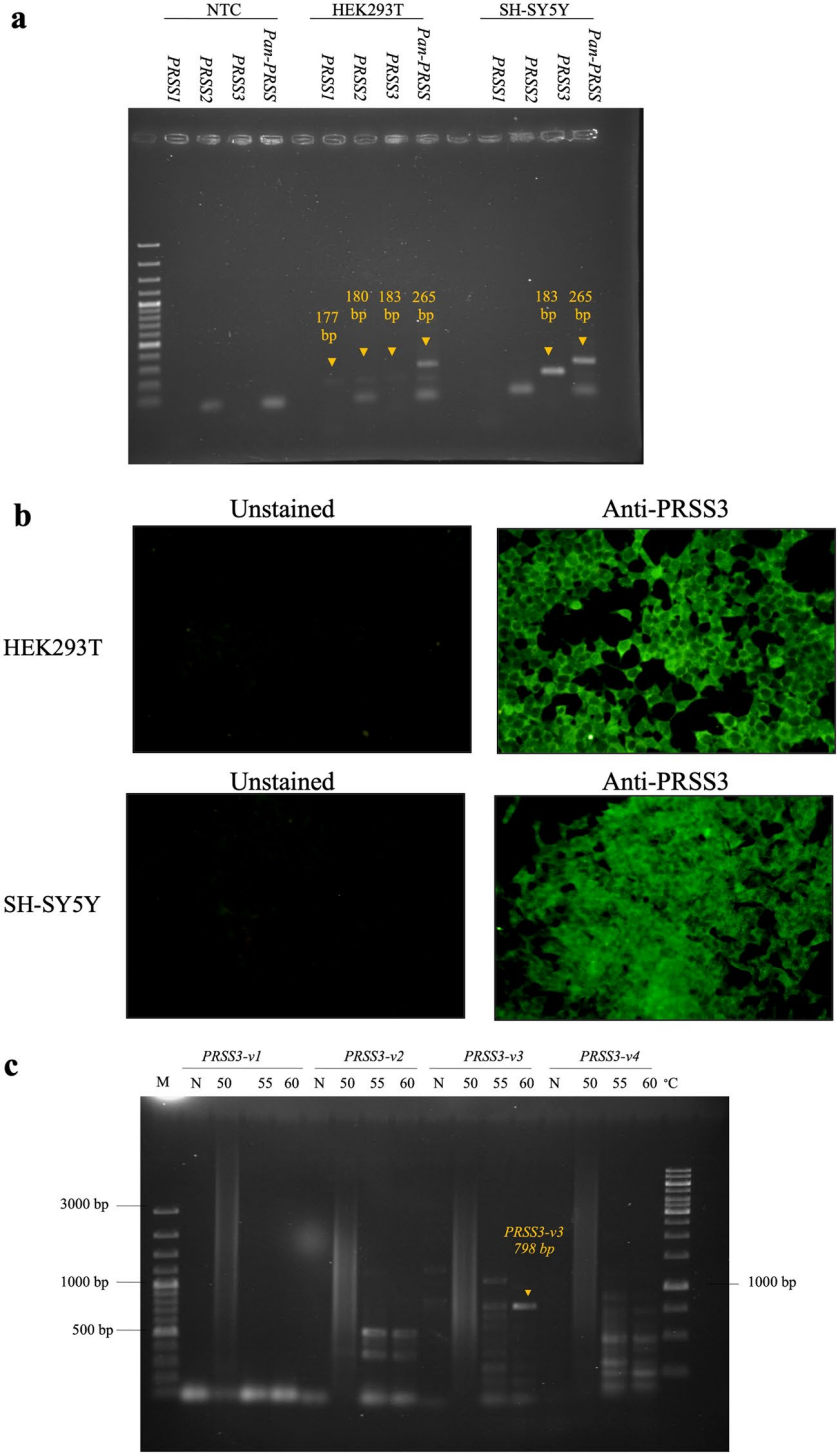
(**a**) Agarose gel electrophoresis of *PRSS1, PRSS2, PRSS3*, and *Pan-PRSS* amplicons determined in HEK293T and SH-SY5Y cells by RT-PCR using specific primers. The sizes of the PCR amplicons of *PRSS1*, *PRSS2*, *PRSS3,* and *Pan-PRSS* are 177, 180, 183, and 265 bp, respectively. (**b**) Micrographs of fluorescent signals from HEK293T and SH-SY5Ycells stained with anti-PRSS3 polyclonal antibody to detect endogenous PRSS3 protein expression. (**c**) Agarose gel electrophoresis of amplicons of full-length PRSS3 transcript variants determined in SH-SY5Y cells by RT-PCR with gradient annealing temperatures. Individual full-length transcript variant of PRSS3 were amplified by RT-PCR using annealing temperatures at 50, 55, and 60 °C. Only PRSS3 transcript variant 3 (*PRSS3-V3*) at the size of 798 bp could be amplified using annealing temperature at 60 °C as indicated by the arrows. Lane M, 100 bp Plus DNA ladder (in base pairs, bp). Lane NTC or N, non-template control.

To clarify which isoform of the human trypsinogen ,i.e. PRSS1 or PRSS3 was expressed in SH-SY5Y cells, the *Pan-PRSS* and isoform-specific primers were used to determine the *PRSS* gene expression by RT-PCR. The expression profiles of human *PRSS* genes including *Pan-PRSS*, *PRSS1*, *PRSS2*, and *PRSS3* were examined. It was found that SH-SY5Y cells strongly expressed *PRSS3,* while *PRSS1* was barely detected (Fig. 3a). The positive control HEK293T expressed all three *PRSS* subtypes. *Pan-PRSS* could be amplified in both SH-SY5H and HEK293T cells. The expression of the PRSS3 protein in SH-SY5Y and HEK293T cells was confirmed by immunofluorescence assay using the commercially available antibody specific to the PRSS3 protein (Fig. 3b). Because *PRSS3* contains 4 transcript variants, the variant identification was performed by RT-PCR using the variant-specific primers to amplify the full-length *PRSS3* transcript variants in SH-SY5Y cells. It was found that only *PRSS3* transcript variant 3 (*PRSS3*-*V3*) could be amplified with its correct size at 798 bp (Fig. 3c). The nucleotide sequence of the suspected *PRSS3*-*V3* was verified by DNA sequencing and nucleotide BLAST (BLASTn) analysis. It was found that the DNA sequencing data showed 100% identity of the sequence to homo sapiens serine protease 3 (*PRSS3*), transcript variant 3 (NCBI reference sequence accession number NM_001197097.3), and homo sapiens protease serine 4 isoform B mRNA, complete cds, accession number AY052783.1. By BLASTx, the insert sequence showed 100% identity of the sequence to serine protease 3 (mesotrypsin), isoform CRA_c [Homo sapiens], accession number EAW58483 deposited in the NCBI database. It was suggested that the EV-A71 3A interacting protein identified in human SH-SY5Y neuronal cells by pull-down assay and LC-MS/MS might be PRSS3 variant 3 (PRSS3-V3) which further validated by immunofluorescence and co-immunoprecipitation assay.

### Demonstration of colocalization of EV-A71 3A with the PRSS3 variant 3 by immunofluorescence assay

**Figure 4:**
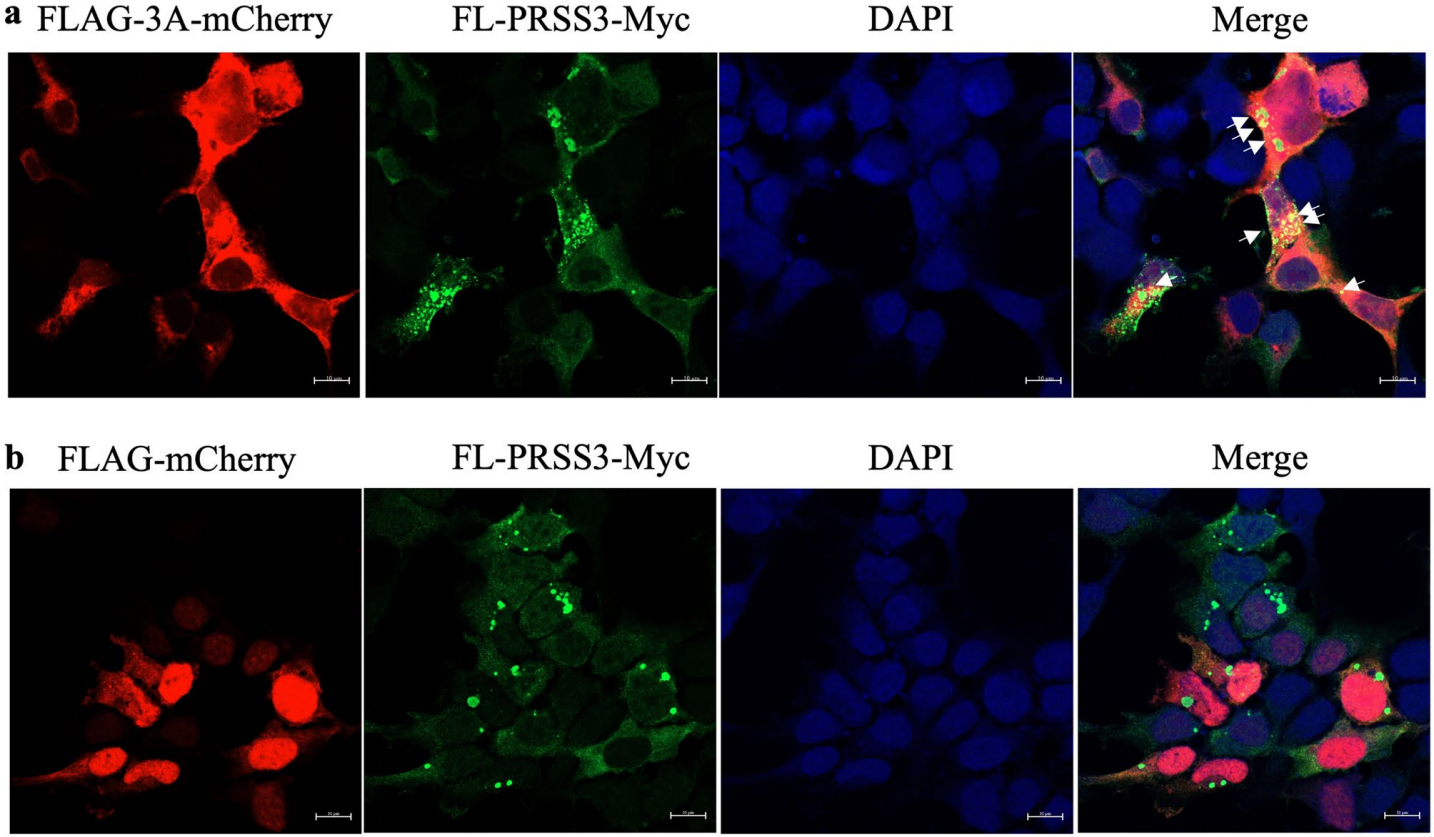
(**a**) Micrographs of HEK293T cells expressed FLAG-3A-mCherry and (**b**) FLAG-mCherry were visualized by confocal microscope (red fluorescence signals). The FL-PRSS3-Myc was immune-stained by specific antibodies (green fluorescence signals). Colocalization of the FLAG-3A-mCherry with FL-PRSS3-Myc was visualized by merging the fluorescence signals which indicated by the arrows and overlaying with DAPI-stained nuclei.

To demonstrate colocalization of EV-A71 3A with PRSS3, the recombinant full-length PRSS3-V3 fused with Myc tag at C-terminus (designated as FL-PRSS3-Myc) and FLAG-3A-mCherry were co-transfected in the HEK293T cells and visualized by immunofluorescence assay using laser confocal microscopy. The cells co-transfected with FL-PRSS3-Myc and FLAG-mCherry served as the negative control. It was found that the FL-PRSS3-Myc spread in the cytosol as puncta and colocalized with the FLAG-3A-mCherry (Fig. 4a). While the FLAG-mCherry found in the nucleus and cytosol did not colocalize with the FL-PRSS3-Myc (Fig. 4b). This finding suggested that EV-A71 3A interacted with PRSS3.

### Confirmation of direct protein–protein binding between EV-A71 3A and the PRSS3 variant 3 by co-immunoprecipitation assay

**Figure 5:**
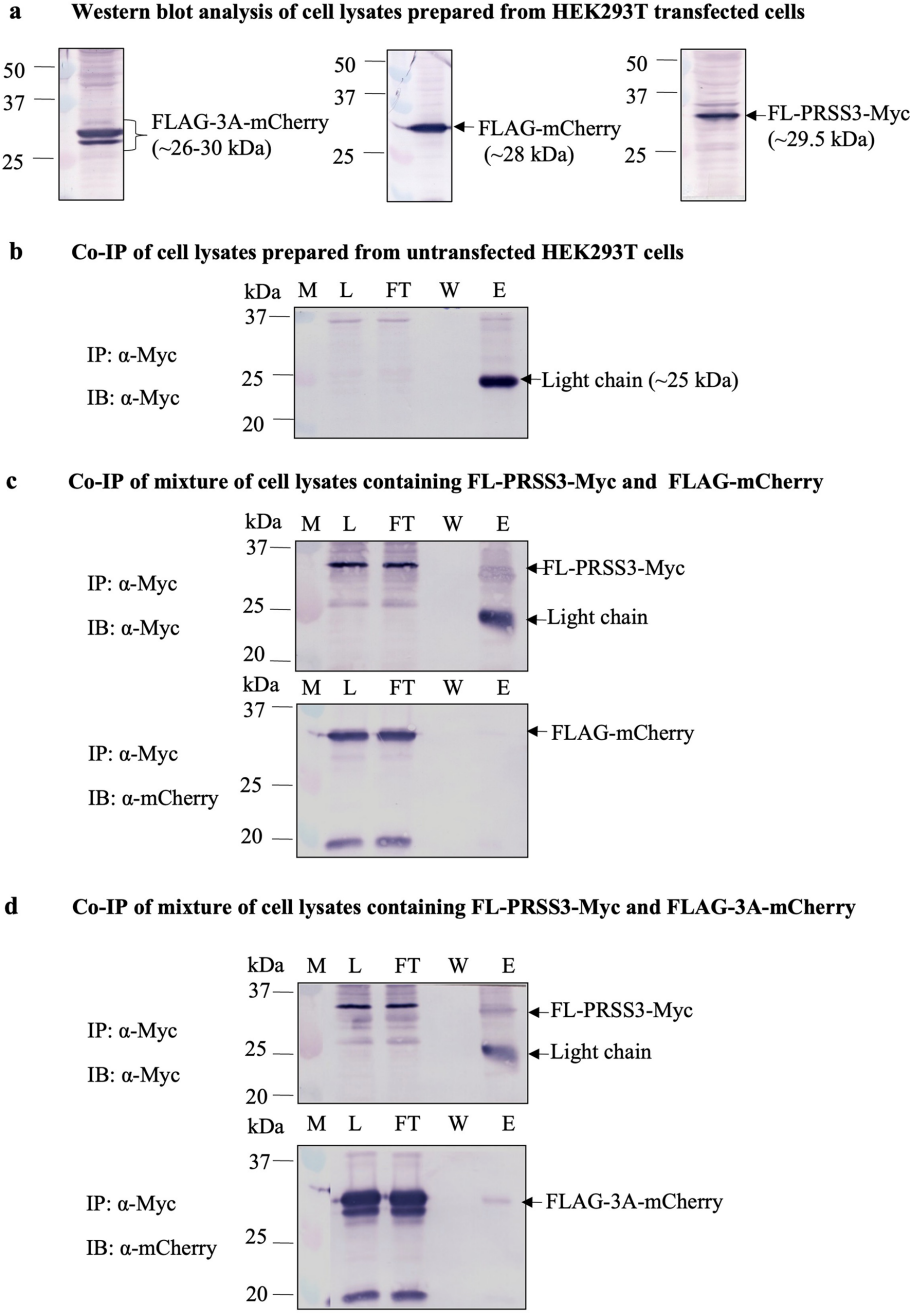
Co-immunoprecipitation (Co-IP) assay for determining the direct protein–protein interaction of FLAG-3A-mCherry and FL-PRSS3-Myc. The FLAG-mCherry served as irrelevant interacting protein. The protein complex was immune-precipitated (IP) by mouse anti-Myc (α-Myc) magnetic beads.The eluted proteins were detected by Western blot analysis (IB) using either mouse α-Myc monoclonal antibody or rabbit anti-mCherry polyclonal antibody (α-mCherry). (**a**) Western blot analysis of expression of FLAG-3A-mCherry, FLAG-mCherry, and FL-PRSS3-Myc. (**b**) Lysates from untransfected HEK293T cells incubated and eluted from the α-Myc magnetic beads. (**c**) Mixtures of FL-PRSS3-Myc and FLAG-mCherry and (**d**) mixtures of FL-PRSS3-Myc and FLAG-3A-mCherry were detected in cell lysate fractions (L) and flow-through fractions (FT) by IB using α-Myc and α-mCherry antibody, respectively. The Co-IP of FLAG-3A-mCherry or FLAG-mCherry in the eluate fractions (E) was determined by IB using α-mCherry. The achievement of Co-IP in (**c**) and (**d**) by immunoprecipitation with the FL-PRSS3-Myc was verified by IP and IB using α-Myc. Light chain of immunoglobulin G (~ 25 kDa) reduced and dissociated from the mouse α-Myc magnetic beads were detected by AP-conjugated goat anti-mouse IgG (H + L) secondary antibody. M, protein standard marker; L, cell lysates; FT, flow-through fractions; W, washed fractions; E, eluate fractions. The original images of (**a–d**) were provided in the Supplementary Fig. 3. The images with adequate length were absent because the blotted membranes were cut prior to hybridization with antibodies.

To confirm the direct interaction of PRSS3 and EV-A71 3A, the FL-PRSS3-Myc and FLAG-3A-mCherry separately expressed in the lysates of HEK293T cells were mixed and pulled down by anti-Myc magnetic beads. The eluate fractions were resolved by SDS-PAGE and the target proteins were examined by Western blot analysis using rabbit anti-mCherry antibody. It was found that only the FLAG-3A-mCherry (~ 26–30 kDa), but not FLAG-mCherry which served as the irrelevant antigen control, was co-immunoprecipitated with FL-PRSS3-Myc protein (Fig. 5). It was confirmed that the EV-A71 3A protein had specifically physical interaction with the PRSS3 variant 3 protein.

### Investigations of the role of PRSS3 in EV-A71 replication by overexpression and gene-silencing approaches

To investigate the role of PRSS3 in EV-A71 replication, the genome replication and the translation of EV-A71 were determined upon overexpression of the FL-PRSS3-Myc and siRNA-mediated gene silencing of endogenous *PRSS3* in the infected cells. Because of the low transfection efficiency of human neuroblastoma SH-SY5Y-cells, HEK293T cell was selected for use as a model for studying the role of PRSS3 in EV-A71 replication. Permissiveness to EV-A71 of the HEK293T cell was validated by CCID50 assay. With EV-A71 viral stock starting at 10^6.125^ CCID50/100 μL, it was found that HEK293T cells gave 10^4.24^ CCID50/100 uL while the viral titers tested in RD cell, a standard cell for EV-A71 enumeration, were corresponding to that of the stock. As a result, HEK293T cell was permissive to EV-A71 infection, but approximately 100 times less strongly than tested in RD cells. Since RD cells require MOI 0.1 for EV-71 infection, therefore, MOI 10 was used for infecting HEK293T cells. The HEK293T cells were used to investigate the role of PRSS3 in EV-A71 replication.

**Figure 6:**
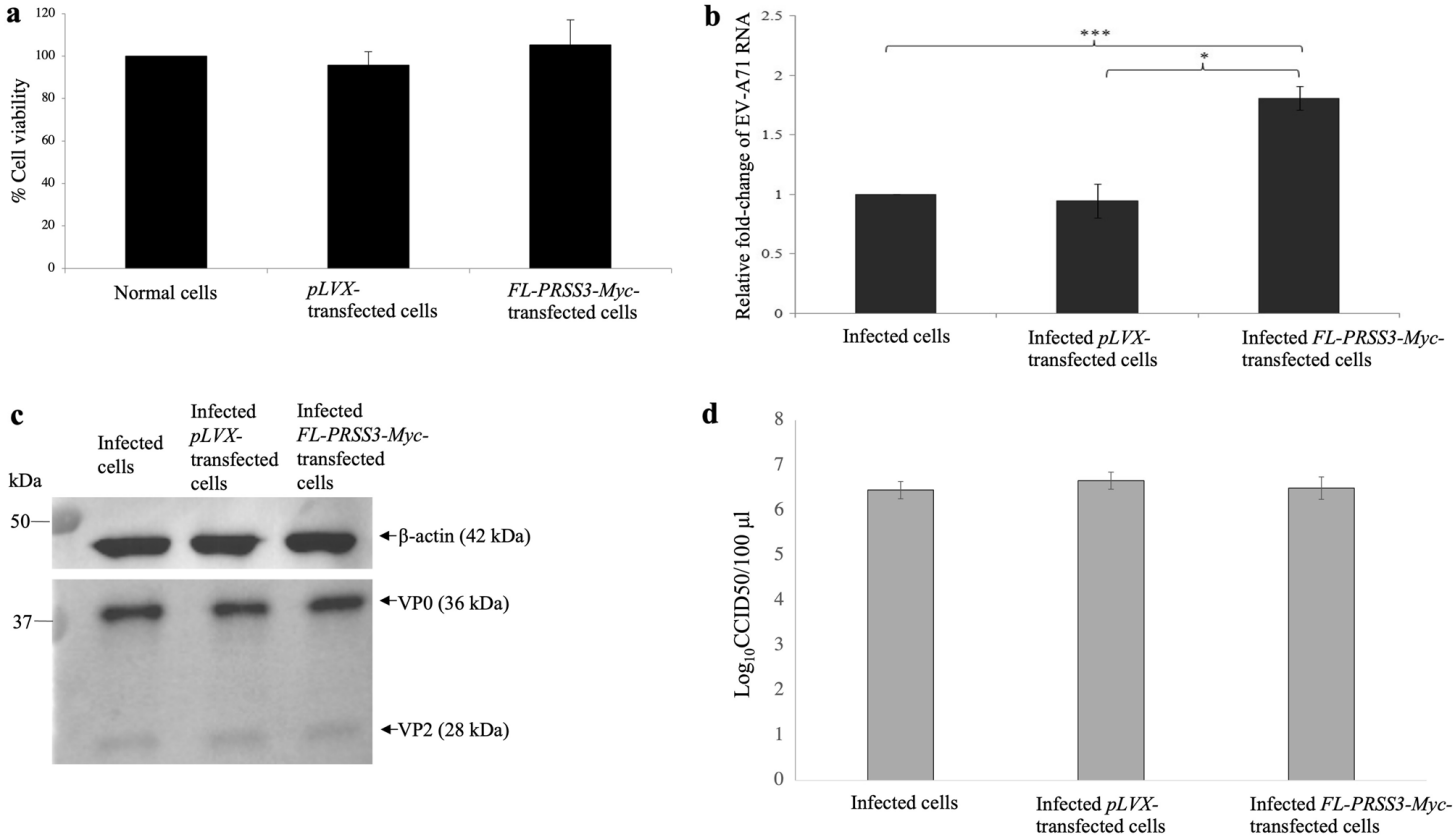
Determination of relative fold-changes of EV-A71 RNA copy numbers by qRT-PCR in response to overexpression of the neuron-derived PRSS3 in *pLVX::FL-PRSS3-Myc*-transfected HEK293T cells. (**a**) Cytotoxicity of the FL-PRSS3-Myc to HEK293T cells was determined by SRB assay. (**b**) The relative fold-changes of EV-A71 RNA copy numbers in response to overexpression of the FL-PRSS3-Myc. The *pLVX::FL-PRSS3-Myc*-transfected HEK293T cells infected with EV-A71 were designated as EV-A71-infected *pLVX::FL-PRSS3-Myc*-transfected cells. Mock transfected and *pLVX-*transfected HEK293T cells infected with EV-A71 served as infection control and empty vector control, respectively. (**c**) Western blot analysis of EV-A71 protein synthesis upon overexpression of the FL-PRSS3-Myc when compared with the control groups. The EV-A71 proteins detected by anti-EV-A71 antibody are VP0 (MW = 36 kDa), pre-cleavage product of VP2, and VP2 (MW = 28 kDa). Human β-actin (MW = 42 kDa) was used for normalization of protein loading. Data were expressed by mean ± SD from three independent experiments in triplicate measurements (N = 9). (**d**) The titers of virus released from cells overexpressed PRSS3, *pLVX-Puro*-transfected- and untransfected cells were determined by CCID50 method. The data was derived from three independent experiments. Statistical analysis was done by One-way ANOVA with post-hoc tests. Levels of statistically significant difference at *p* < 0.05 and *p* < 0.001 were indicated by * and ***, respectively. The original blots of (**c**) were shown in Supplementary Fig. 2.

Before assessing the effect of over-expression of the recombinant FL-PRSS3-Myc on EV-A71 infection, it was necessary to ensure that FL-PRSS3-Myc did not elicit cytotoxicity to the transfected HEK293T cells (Fig. 6a). Upon the EV-A71 infection, it was found that the overexpression of the FL-PRSS3-Myc protein caused a nearly two-fold increase in viral RNA replication compared to the *pLVX-Puro*-transfected- and untransfected cells (Fig. 6b). At the same time, the viral RNA replication in *pLVX-Puro*-transfected HEK293T cells was not significantly different from the untransfected cells. However, the expression levels of VP0 and VP2 proteins among them were not different (Fig. 6c). This might be due to end-point measurement of an expression of the viral proteins performed in this study. The expression of the viral proteins should be measured at different time-points post-infection to determine the dynamic of expression. The titers of virus released from cells overexpressed PRSS3, *pLVX-Puro*-transfected- and untransfected cells did not show a different in an order of magnitude, which were 10^6.5^ ± 10^0.25^, 10^6.67^ ± 10^0.19^, and 10^6.45^ ± 10^0.19^ CCID50/100 μL, respectively (Fig. 6d). It suggested that overexpression of the neuron-derived PRSS3 marginally enhanced the genome replication of EV-A71 but did not affect the viral protein synthesis and virion maturation.

**Figure 7:**
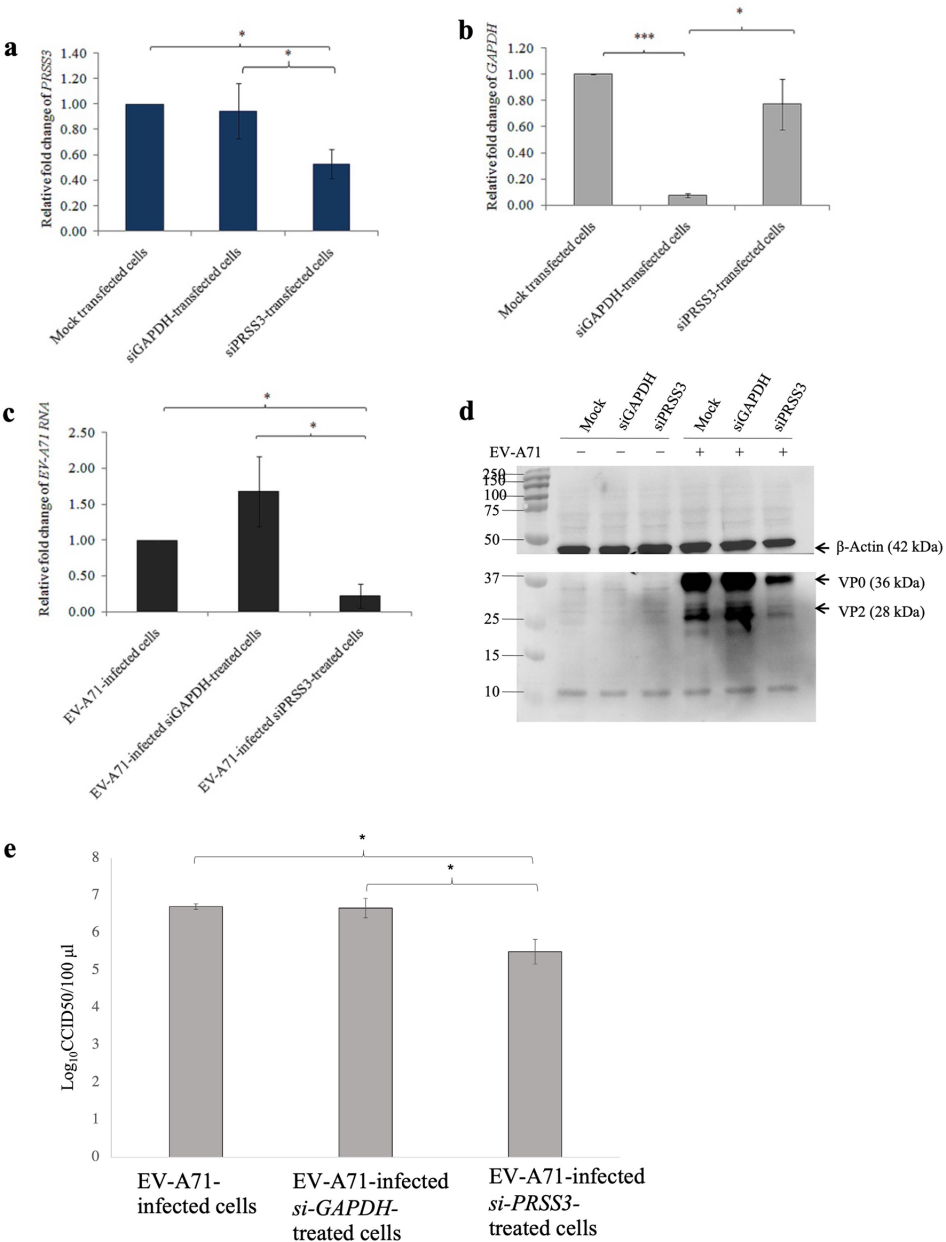
Determination of relative fold-changes of EV-A71 RNA copy numbers by qRT-PCR in response to the knockdown of *PRSS3*. (**a**) Fold-changes of gene expression of *PRSS3* and (**b**) *GAPDH* in the siRNAs-transfected non-infected cells were relative to the mock transfected non-infected cells. (**c**) Relative fold-changes of EV-A71 RNA copy numbers in *siPRSS3*-transfected HEK293T cells infected with EV-A71 when compared with *siGAPDH-*transfected- and untransfected infected cells. The qRT-PCR data were expressed by mean ± SD from three independent experiments in triplicate measurements (N = 9). (**d**) Western blot analysis of EV-A71 protein synthesis upon siRNA-mediated gene silencing of *PRSS3* when compared with the control groups*.* The EV-A71 proteins detected by anti-EV-A71 antibody are VP0 (MW = 36 kDa), pre-cleavage product of VP2, and VP2 (MW = 28 kDa). Human β-actin (MW = 42 kDa) was used for normalization of protein loading. (**e**) The titers of virus released from *PRSS3*-silenced cells, *si*-*GAPDH* transfected- and untransfected cells were determined by CCID50 method. The data was derived from triplicate experiments. Statistical analysis was done by One-way ANOVA with post- hoc tests. Levels of statistically significant difference at *p* < 0.05 and *p* < 0.001 were indicated by * and ***, respectively. The original blots of (**d**) were shown in Supplementary Fig. 2.

To determine whether *PRSS3* expression could be silenced by siRNA, HEK293T cells were transfected with either three-pooled siRNAs targeting *PRSS3* (*siPRSS3*) or *siGAPDH* which served as a control for siRNA transfection and irrelevant gene knockdown. It was found that the silencing of *PRSS3* (Fig. 7a) and *GAPDH* (Fig. 7b) using siRNA was successful as determined by qRT-PCR. The protein levels of PRSS3 and GAPDH were also reduced in the *siPRSS3-*treated- and *siGAPDH*-treated cells, fold-change at 0.86 and 0.6 relative to mock cells, respectively (Supplementary Fig. 4a). The fold-change of expression levels of GAPDH protein of the *siGAPDH*-treated- and *siPRSS3-*treated cells was 0.6 and 0.92 relative to mock, respectively (Supplementary Fig. 4b). The results were representative data from one of three replicate experiments. The silencing of the *GAPDH* did not affect the expression of the *PRSS3* which allowed it to be eligible to serve as irrelevant gene knockdown control. The gene knockdown did not cause cytotoxicity to the transfected cells as the *siPRSS3*- and *siGAPDH*-treated cells elicited 100.22 ± 12.25% and 102.66 ± 6.95% cell viability relative to normal cells, respectively. Next, to assess the role of PRSS3 in EV-A71 replication, HEK293T cells were transfected with *siPRSS3* or *siGAPDH* followed by EV-A71 infection at MOI 10. It was found that knockdown of *GAPDH* did not significantly affect EV-A71 replication while *PRSS3* knockdown resulted in reduction of EV-A71 RNA (Fig. 7c). EV-A71 proteins from the same samples were also detected by Western blot analysis using anti-EV-A71 antibody specific to the viral capsid protein VP0 and its cleaved form, VP2. It was found that the levels of the translated EV-A71 proteins decreased when compared to the controls (Fig. 7d). Using the Image Lab Program to estimate the protein intensity of VP0 showed that the protein levels in the PRSS3 knockdown sample was lesser than those of EV-A71-infected and EV-A71-infected *siGAPDH*-treated cells for 2.8 and 3.1 times, respectively. The intensity of VP0 protein band in EV-A71-infected *siGAPDH*-treated cells was 1.13 times of those of EV-A71-infected cells. Thus, silencing of *PRSS3* resulted in reduction of the EV-A71 genome translation. Consistently, the titers of virus released from *PRSS3*-silenced cells showed a different in an order of magnitude, which were 10^5.5^ ± 10^0.33^ CCID50/100 μL, comparing to *siGAPDH*-transfected- and untransfected cells, which were 10^6.67^ ± 10^0.26^ and 10^6.71^ ± 10^0.07^ CCID50/100 μL, respectively (Fig. 7e). Taken together, it could be concluded that EV-A71 3A protein directly interacted with PRSS3 variant 3 protein in human neuronal cells and thus PRSS3 plays the essential role in the EV-A71 replication.

## Discussion

Enterovirus A71 (EV-A71) infection can cause hand, foot, and mouth disease (HFMD) and culminate in neurological complications such as meningitis, acute flaccid paralysis, neurologic cardiopulmonary failure and even death^11^. So far, there is no specific treatment or vaccine for EV-A71 infection. Understanding the viral neuropathogenesis might provide the strategies to prevent or mitigate deleterious effects from the virus. Enterovirus nonstructural proteins, 2B-2C, and 3A-3D, play several roles in the viral replication cycle. To establish the successful infection, the virus must produce several viral progenies throughout the time of infection, and, at the same time, the virus must evade or protect itself from recognition by intracellular immune sensing. Enterovirus has evolved the strategies to survive in the host cell by reorganizing the host cellular proteins and lipids to build up the platforms for negative-sense viral RNA synthesis and viral assembly termed as replication complexes (RCs) or replication organelles (ROs)^16,17^. It has been reported that enterovirus nonstructural 3A protein (3A) is the key player which mediates the RO formation^19–21^. The virally modified platforms promoted the negative-sense viral RNA replication by concentrating the co-opted host factors and viral proteins and provided the proper milieu for viral synthesis. Moreover, ROs served as the shelter, protecting the double-stranded RNA intermediate from the host immune detection. The composition of ROs is seemingly diverse depending on the host organelle donor, the preferential location of RO formation, and the host cell that the virus infects. It was reported that enterovirus ROs are originated from ER and trans-Golgi network upon infection^26^.

Detection of EV-A71 antigens and RNA in CNS tissue sections from encephalitis patients showed exclusive localization of the virus in neuronal cells at the inflamed areas. These areas included spinal cord, medulla, pons, midbrain, hypothalamus, dentate nucleus area of cerebellum, and motor cortex^27^. The localization of EV-A71 in human neuronal cells corresponded to the studies in the animal models^28,29^. In vitro study conducted in neuronal cells might be developed to scrutinize EV-A71 neuropathogenesis. Even though the use of primary cells derived from CNS tissues might provide an accurate recapitulation of the neuronal cell properties in vivo, the isolation and culture of the primary cells is challenging as well as the ethical approval is also required. In addition, the primary cells are generally known as difficult-to-transfect cells. To overcome the limitations, human neuroblastoma cell lines have been used not only for studying neurological disorders but also for neurotropic virus infection. Two commonly used human neuronal-like cell lines are SH-SY5Y and SK-N-MC (also known as HTB-10). Human SH-SY5Y is a thrice-subcloned cell line derived from the parental SK-N-SH neuroblastoma cell line originated from metastatic cells in bone marrow, while SK-N-MC neuroepithelioma is originated from supra-orbital region. Determination of the neuronal cell marker genes by RNA-sequencing showed higher expression levels of the genes in SH-SY5Y than those of SK-N-MC, indicating high neuronal-like properties of SH-SY5Y^30^. Human SH-SY5Y cells have been used in many studies in both neurodegenerative diseases such as Parkinson's disease and neurotropic virus infection such as enterovirus A71 (EV-A71), enterovirus D68 (EV-D68), poliovirus, rabies virus, Japanese encephalitis virus (JEV), zika virus (ZIKV), dengue virus (DENV), and herpes simplex virus infections^30–37^. Importantly, SH-SY5Y cells expressed SCARB2, the entry receptor for EV-A71^38^. To date, neuropathogenesis of enterovirus A71 is poorly understood. The role of EV-A71 3A protein in infected neuronal cells remains unknown. Taken together, in this study, the neuron-derived host factor which interacted with the EV-A71 3A protein was identified and its role in the viral replication was explored. The direct protein–protein interaction between homo sapiens serine protease 3 (PRSS3), the PRSS3 transcript variant 3 or mesotrypsin, derived from the human neuroblastoma SH-SY5Y cell and the recombinant EV-A71 3A protein (FLAG-3A-mCherry) was confirmed. This suggested that the 3A protein might recruit this host cellular protein to serve certain aspects in the viral replication in human neuronal cells. Due to the low transfection efficiency of SH-SY5Y cells, the role of neuron-derived PRSS3 in EV-A71 replication was subsequently studied in the susceptible HEK293T cells. Overexpression of the neuron-derived recombinant PRSS3 variant 3 marginally enhanced the genome replication of EV-A71 but did not affect the viral protein synthesis and virion maturation. The virus replication seemed to reach steady-state at the time-point determined. This might be due to influence of the endogenous PRSS3 level which did not allow us to determine the effect of overexpression of exogenous PRSS3 on the viral protein synthesis and maturation. Moreover, the PRSS3 overexpression might be in excess of amount of its ligand (3A or polyprotein of EV-A71), which the binding of them might take place on the replication organelles. It has been shown previously that the binding of an excess of the ligand with its receptor on the cell surface leads to a steric hindrance effect in which the bound receptors could exclude receptor binding at the neighboring ligand sites^39^. Hence, the PRSS3 overexpression did not any show the additional effect on viral replication. The involvement of PRSS3 in RNA replication and genome translation was also confirmed by *PRSS3* gene knockdown using siRNA. Silencing of *GAPDH* served as irrelevant gene knockdown showed marginally increment in relative RNA levels of the EV-A71. It is possibly due to the involvement of GAPDH in the IFN-γ and NO pathways that regulate viral replication as previously reported in coronaviruses^40^. However, this observation warrants further experimental validations. In our preliminary data, it was found that EV-A71 infection did not affect the expression level of *PRSS3* mRNA at 12- and 24 h post-infection (data not shown). The molecular mechanism of PRSS3 and 3A viral protein in EV-A71 pathogenesis is still unclear and requires further investigations.

Trypsinogen is the proactive precursor of trypsin which is one of the important serine proteases. Human trypsinogens have been classified according to the distinct isoelectric points (pI) into 3 major isoforms: PRSS1 (cationic trypsinogen), PRSS2 (anionic trypsinogen), and PRSS3 (mesotrypinogen)^41^. They were initially isolated from human pancreatic juice. Later, it was found that trypsinogens are also expressed by several other tissues as well as the central nervous system (CNS)^42,43^. Human trypsinogens are increasingly implicated with virus infections. For examples, human PRSS1 (cationic trypsinogen) is one of the EV-A71 IRES-associated proteins in human glioblastoma T98G cells and the cleavage of hemagglutinin (HA) by activated human trypsinogen (PRSS3) enhanced influenza A virus infection^44,45^. Since the main function of the viral 3A is to recruit the host cellular proteins or lipids to generate the replication organelles (ROs), the interacting counterparts of 3A might provide certain benefits for the viral replication. One of the reported 3A-interacting partners is cholesterol which regulates the membrane-dependent polyprotein processing of viral 3CD protease and facilitates the viral replication^17^. Even though the virus has its own proteases, it has been found that calpains, the host cellular papain-like cysteine proteases which are expressed as proenzyme in cytoplasm and activated by an increasing of intracellular Ca^2+^, could cleave polyprotein of enterovirus B at VP3-VP1 interface in vitro^46^. Despite being the proactive enzyme, autoactivation of trypsinogens was reported in both PRSS1 and PRSS2^47,48^. The trypsin cleavage sites of the PRSS on the amino acid sequence of EV-A71 polyprotein were predicted by PeptideCutter program (Supplementary Data S1). It was shown that there are 194 tentative trypsin cleavage sites on the EV-A71 polyprotein. Interestingly, the predicted trypsin cleavage site at position 69 corresponded to VP4-VP2 junction, so-called VP0 cleavage site^49^. In viral assembly, the viral capsid precursor P1 is primarily cleaved by viral 3CD^pro^ into VP0, VP1, and VP3 which are assembled into 5S protomer. Five protomers are then combined to form 14S pentamer. The viral 2C^ATPase^ recruits the 14S pentamers to replication organelles (ROs). To achieve the virion maturation, VP0 is cleaved into VP2 and VP4 which change the capsid conformation. It has been found that 2A^pro^ and 3C^pro^ proteins of EV A71 did not participate in VP0 cleavage^49^. So far, the speculated mechanism of cleavage of VP0 is RNA-dependent autocatalytic action. Since viral proteases also play important roles at ROs to facilitate the viral replication, the cleavage sites of both EV-A71 proteases; 2A^pro^ and 3C^pro^, on the 3A-interacting PRSS3 protein were evaluated. It was found that there is one cleavage site of 2A^pro^ at amino acid position 164 and two cleavage sites of 3C^pro^ at position 182 and 225 (Supplementary Data S2). Thus, it could be postulated that PRSS3 that were recruited to ROs by EV-A71 3A protein might be cleaved by the viral proteases to get maturation and then turned to facilitate the viral replication. At the ROs, the mature PRSS3 might be involved with membrane-dependent viral polyprotein processing or maturation of viral assembly by cleavage of VP0. These notions and the postulated mechanism of PRSS3 in facilitating the EV-A71 replication remain to decipher. Serine proteases are involved in many biological processes such as proteolytic processing of the protein, food digestion, signal transduction, and immune response^50^. In the CNS, it has been reported that serine proteases are widely expressed and play diverse roles including structural plasticity, neural development, regulation of neuronal survival, neurodegeneration, and neuroregeneration^51–53^. For brain trypsinogens, the study in rat tissue model showed that PRSS2 (anionic trypsinogen) secreted by activated microglial cells promoted generation of white matter neurons^54^. Trypsinogens function as a signaling agent by cleaving extracellular N-terminus of protease-activated receptors (PARs) resulting in activation of signal cascades via MAPK/ERK pathway. This leaded to the promotion of cell growth and survival. Further, treatment of primary neuronal cells with trypsin rendered the protective effect against glutamate excitoxicity^55^. Hence, trypsinogens exert the crucial physiological functions in CNS. Trypsinogens are secreted proteins, therefore, destined to be transported via the cellular secretory pathway. In this study, the interaction between PRSS3 trypsinogen and EV-71 3A might cause retention of the protein at the viral replication organelles (ROs). Importantly, one of the pathological features of neurodegenerative diseases such as Alzheimer's disease (AD), Amyotrophic lateral sclerosis (ALS), and Parkinson’s disease (PD) is the intracellular protein toxicity caused by accumulation or aberrant localization of the toxic protein^56–58^. Therefore, retention of the PRSS3 trypsinogen in the human neuronal cells might be one of the pathological mechanisms in EV-A71 infection. This notion warrants further investigations. The gained knowledge from this study provided a better understanding about the viral replication in the neuronal cells and offered the promising host candidate to be a target for further investigations to pave the way for generation of EV-A71 specific treatment and development of biomarker for prognosis of the disease.

## Methods

### Cells and virus

Human embryonic kidney 293 T (Lenti-X HEK293T) cells, rhabdomyosarcoma (RD) cells (JCRB9072), and neuroblastoma SH-SY5Y cells (ATCC, CRL-2266) maintained in our laboratory were routinely cultured at 37 °C and 5% CO_2_. HEK293T and RD cells were cultured in Dulbecco’s modified Eagle’s medium (DMEM, Biochrom, Cambridge, UK) and SH-SY5Y cells were cultured in DMEM/F12 (Gibco, NY, USA). Both media were supplemented with 10% fetal bovine serum, L-glutamine, and penicillin/streptomycin antibiotics (Gibco, NY, USA). The EV-A71 viral stock (Thai clinical isolate, genotype B5 designated as TUCU001/B5 isolate) was propagated in 90–95% confluence of RD cell monolayer. The viral titers were determined by CCID50 method as described previously^59^.

### Antibodies and reagents

Mouse anti-FLAG M2 magnetic beads and mouse monoclonal anti-FLAG M2 antibody were purchased from Sigma-Aldrich (USA). Rabbit anti-mCherry polyclonal antibody was obtained from Biovision (USA). Mouse monoclonal anti-cMyc antibody and mouse anti-enterovirus 71 VP2 antibody were from Bio-Rad (USA). Mouse anti-Myc-tag monoclonal antibody (clone 9B11) magnetic beads were from Cell Signaling Technology (USA). Rabbit anti-PRSS3 polyclonal antibody (clone PA-5-23991 and PA5-11407 for immunofluorescence assay Western blot analysis, respectively) and AlexaFluor488 goat anti-mouse IgG (H + L) were from Thermo Fisher Scientific (USA). Mouse anti-ß-actin antibody was purchased from Affinity Biosciences (USA). Rabbit anti-GAPDH (clone 14C10) monoclonal antibody was purchased from Cell signaling technology. Alkaline phosphatase (AP)-conjugated goat anti-mouse IgG (H + L) antibody and KPL BCIP/NBT substrate solution were from SeraCare (USA). Horseradish peroxidase (HRP)-conjugated goat anti-rabbit IgG (H + L) antibody and enhanced chemiluminescence (ECL) detection system (Clarity Max Western ECL substrate) was form Bio-Rad. DynaMag-Spin magnet was brought from Life Technologies (UK) .

### Construction of recombinant plasmids and transfection

Coding sequences of epitope tag *FLAG* and red monomeric fluorescent protein *mCherry* were molecularly fused in-frame with EV-A71 nonstructural *3A* coding sequence at 5′- and at 3′-end, respectively. To generate *FLAG-3A-mCherry* gene fusion, two overlapping DNA sequences including *EcoRI-FLAG-3A-mCherry* and *3A-mCherry-XbaI* were amplified separately to subsequently generate the full-length fragment of *EcoRI-FLAG-3A- mCherry-XbaI* using overlap extension polymerase chain reaction. The *EcoRI-FLAG-3A-mCherry*was amplified from complementary DNA (cDNA) of EV-A71 3A inserted in recombinant pLVX-Puro plasmid (Clontech) by Outer1-MamNS3A forward primer (5′-*GAATTC***ATGGATTACAAGGATGACGATGACAAG**GGCCCGCCCAAGTTC-3′, the italic letters are *EcoR*I restriction site, the bold letters are *FLAG* coding sequence); and Inner1-mCherry-3A reverse primer (5′-**GCCCCTTGCTCACCAT**TTGAAACCCCGCAAAGAG -3′, the bold letters are the first 16 nucleotides of *mCherry* coding sequence). The *3A-mCherry-XbaI* was amplified from cDNA of mCherry inserted in pLV-mCherry (a gift from PantelisTsoulfas, Addgene plasmid #36,084; http://n2t.net/ addgene:36,084; RRID: Addgene_36,084) by Inner2-3A-mCherry forward primer (5′-**AGCTCTTTGCGGGGTTTCAA**ATGGTGAGCAAGGGCGAGGAGGATAACATG-3′, the bold letters are the last 20 nucleotides of *3A* coding sequence) and Outer2-mCherry reverse primer (5′-*TCTAGA*TTACTTGTACAGCTCGTCCAT-3′, the italic letters are *Xba*I restriction site). The thermocycling program was 95 °C for 5 min followed by 30 cycles of 95 °C for 30 s, 68 °C for 30 s, 72 °C for30 seconds, and finally 72 °C for 2 min. The agarose gel-purified *EcoRI-FLAG-3A-mCherry* and *3A-mCherry-XbaI* DNA fragments were mixed with PCR reaction mixture and heated at 95 °C for 5 min to denature the double-strands of the DNA templates. Then, the temperature was brought down gradually from 95 to 45 °C with decrement rate at − 1 °C per minute for 2 min. In this step, the *EcoRI-FLAG-3A-mCherry* and *3A-mCherry-XbaI* were slowly annealed at the overlapping sites and their overhang termini were extended by filling with deoxynucleotides at 68 °C for 1 min. Consequently, the full-length DNA template of *EcoRI-FLAG-3A-mCherry-XbaI* coding sequences was generated. Immediately, the thermal cycle was paused and Outer1-MamNS3A forward primer and Outer2-mCherry reverse primer were added into the reaction mixture. Afterward, the thermal cycle was resumed to amplify *EcoRI-FLAG-3A-mCherry-XbaI* coding sequences by thermocycling program of 30 cycles of 95 °C for 30 s, 55 °C for 1 min, and 68 °C for 1 min with final extension at 68 °C for 3 min. To generate the full-length sequence of *PRSS3* transcript variant 3 (*PRSS3-V3*) in-frame with *Myc* coding sequence at 3′-end (*FL-PRSS3-Myc*), cDNA of *PRSS3-V3* was amplified from RNA extracted from SH-SY5Y cells using 1-prss3v3 forward primer (5′-*CTCGAG*ATGCACATGAGAGAGACAAGT-3′, the italic letters are *Xho*I restriction site, and 783-Myc-prss3v3 reverse primer (5′-*TCTAGA*TTA**CAGATCCTCTTCTGAGATGAGTTTCTGCTC**GCTGTTGGCAGCGATGGTGTC-3′, the italic letters are *Xba*I restriction site, the bold letters are *Myc* coding sequence). The thermocycling program was 95 °C for 10 min followed by 30 cycles of 95 °C for 30 s, 62 °C for 45 s, and 72 °C for 1 min and finally 72 °C, for 3 min. To construct recombinant plasmid DNA carrying coding sequence of *FLAG-mCherry*, *mCherry* coding sequence was amplified from the *pLV-mCherry* using *FLAG-mCherry* forward primer (5′-*CTCGAG***ATGGATTACAAGGATGACGATGACAAG**GTGAGCAAGGGCGAGGAGGAT-3′, the italic letters are *Xho*I restriction site, the bold letters are *FLAG* coding sequence) and the Outer2-mCherry reverse primer. The *FLAG-3A-mCherry*, *FL-PRSS3-Myc*, and *FLAG-mCherry* coding sequences were ligated into cloning vectors and subsequently subcloned into mammalian expression vector pLVX-Puro using the respective restriction sites, namely *pLVX-Puro::FLAG-3A-mCherry, pLVX-Puro::FL-PRSS3-Myc*, and *pLVX-Puro::FLAG-mCherry*, respectively.

Transient transfection was performed on cell monolayers reached 70%-80% confluence using Lipofectamine 3000 reagents according to the manufacturer’s instructions (Thermo Fisher Scientific, CA, USA). In a 24-well culture plate, HEK 293 T cells were seeded at a density of 5 × 10^4^ cells/well and SH-SY5Y cells were seeded at a density 1 × 10^5^ cells/well. In a 6-well culture plate, HEK 293 T cells were seeded at a density of 3 × 10^5^ cells/well and SH-SY5Y cells were seeded at a density1 × 10^6^ cells/well.

### Pull-down assay and protein identification by LC MS/MS

To identify EV-A71 3A-interacting protein, SH-SY5Y cell monolayers grown in 2 wells of a 6-well plate were transfected with *pLVX-Puro::FLAG-3A-mCherry*. SH-SY5Y cell monolayers were also transfected with *pLVX::FLAG-mCherry* to serve as pull-down background control. After 24 h of transfection, cells were harvested and washed with 1 × PBS. To prepare cell lysate, cells were resuspended in 1 mL of lysis buffer (0.5% Triton X-100, 10% glycerol, 25 mM Na-HEPES, 75 mM NaCl, and 0.5 mM EDTA, pH 7.4 ) containing protease inhibitor cocktail and mixed thoroughly by pipetting. Then, the resuspended cells were incubated at room temperature on a roller shaker for 1 h and completely lysed by ultrasonication. The homogenates were centrifuged to separate cellular debris and cell lysates were collected and kept on ice. To pull down the EV-A71 3A interacting proteins, mouse anti-FLAG M2 magnetic beads were used. Magnetic beads were washed with lysis buffer and separated on DynaMag-Spin magnet for 3 times. Then, the cell lysates prepared from FLAG-3A-mCherry transfected SH-SY5Y cells and pull-down background control were incubated with the equilibrated magnetic beads with rotation at 4 °C for 2 h. The magnetic beads were captured on DynaMag-Spin magnet, the unbound fraction was collected. The beads were washed 3 times with wash buffer 1 (lysis buffer containing 0.5% Triton X-100 and 5% glycerol) followed by washing 3 times with wash buffer 2 (lysis buffer containing 5% glycerol). The last wash fraction was collected. To elute the bead-bound proteins, elution buffer (5 mM Na-HEPES, 100 mM NaCl, pH 7.4) containing 3 × FLAG peptide (Sigma Aldrich, USA) was mixed with the beads and incubated for 30 min on a rotating platform. The elution step was done repeatedly two more times. The concentration of each protein fraction was measured using Bradford protein assay. The loading amount of the protein fractions per well was normalized and resolved in 12% SDS-PAGE. The bands of the resolved proteins were double-stained with Coomassie brilliant blue and silver dye (ProteoSilver Silver Stain Kit, Sigma-Aldrich) following the manufacturer's instruction to enhance the sensitivity of the visualization. The protein bands of the pull-down of the FLAG-3A-mCherry transfected SH-SY5Y cells were compared to the protein bands in the pull-down background control and in the protein background controls including wash fractions, cell lysates prepared from untransfected SH-SY5Y cells, and cell lysates prepared from SH-SY5Y cells transfected with *pLV-mCherry*. The distinguishable protein bands were excised from the SDS-PAGE gel. The gel plugs were sent to service unit of Proteomics International, Australia, for protein identification by LC-MS/MS. The presence of FLAG-3A-mCherry and FLAG-mCherry proteins in pull-down fractions was validated by Western blot analysis using mouse monoclonal anti-FLAG M2 antibody followed by alkaline phosphatase (AP)-conjugated goat anti-mouse IgG (H + L) antibody. The reactive bands were visualized by adding KPL BCIP/NBT chromogenic substrate solution.

To de-stain the silver dye, the gel pieces of silver-stained bands were incubated with a 1:1 solution of 30 mM potassium ferricyanide and 100 mM sodium thiosulfate for 10 min for 2 times. Then, the gel pieces were washed for 2 times with water for 1 h and 25 mM ammonium bicarbonate in 50:50 acetonitrile (ACN):water for 10 min. The de-stained and washed gel pieces were vacuum-dried. To perform trypsin digestion, the gel pieces were incubated with trypsin digest solution (12.5 mg/mL trypsin, 25 mM ammonium bicarbonate) at 37 °C for overnight. The digested peptides were extracted by incubating with ACN containing 1% trifluoroacetic acid (TFA) for 20 min for 2 times. The digested peptides were analyzed by LC-MS analysis using the Agilent 1260 Infinity HPLC system coupled to an Agilent 1260 Chip Cube Nanospray interface on an Agilent 6540 mass spectrometer. Peptides were loaded onto a ProtID-Chip-150 C18 column (Agilent) and separated with a linear gradient of water/acetonitrile/0.1% formic acid (v/v). Spectra were analyzed to identify proteins of interest using Mascot sequence matching software (Matrix Science) with MSPnr100 database. Protein identification was performed based on statistically significant Mascot score (*p* < 0.05).

### Profiling of transcriptional variants of PRSS in SH-SY5Y cells

The gene expression profiles of PRSS human trypsinogens pulled down with EV-A71 3A and identified by LC-MS/MS were determined by conventional reverse- transcription PCR (RT-PCR) using Pan-PRSS and subtype-specific primers previously described by Hayashi et al*.* as listed in Table 2. cDNA was synthesized from RNA extracted from SH-SY5Y cells and amplified using thermal cycling program as follows; 95 °C for 5 min, 30 cycles of 95 °C for 30 s, 57 °C for 45 s, 72 °C for 40 s and finally 72 °C for 2 min. The expression of the specific type of PRSS in SH-SY5Y cells was confirmed by immunofluorescence assay. The SH-SY5Y cell monolayers grown on a round glass coverslip placed in a 24-well plate were fixed with 4% paraformaldehyde, blocked with 5% bovine serum albumin, and incubated with type-specific anti-PRSS primary antibody followed by Alexa Fluor 488 goat anti-mouse IgG (H + L). To visualize, the round glass coverslips were placed upside down on a glass slide with a small drop of 80% glycerol, sealed, and observed under fluorescence microscope. The transcriptional variants of the specific PRSS expressed in human SH-SY5Y neuronal cells were identified by RT-PCR and DNA sequence analysis. The full-length cDNA of PRSS was amplified by PCR using variant-specific primers (Table 2). PCR protocol was as follows; 95 °C for 5 min, 30 cycles of 95 °C for 30 s, gradient temperature at 50–60 °C for 45 s, and 72 °C for 1 min. The DNA sequencing data of full-length *PRSS* were verified by bioinformatic analysis.

**Table 2:** Primers used for determination of PRSS gene expression.

Primer name	5′ to 3′ sequence	Reference
**Pan-PRSS**		
Pan-PRSS forward	GCCAAGATCATCCGCCACCC	^45^
Pan-PRSS reverse	TCTTTCCAGGGTAGGAGGCTT	
**Subtype-specific**		
PRSS1 forward	CCACCCCCAATACGACAGGAA	^45^
PRSS1 reverse	TAGTCGGCGCCAGAGCTCGC	
PRSS2 forward	CCACCCCAAATACAACAGCCG	
PRSS2 reverse	GGGTAGTCGGCACCAGAACTCAG	
PRSS3 forward	CGCCACCCTAAATACAACAGGGA	
PRSS3 reverse	TGGGTAGTCAGCACCAAAGCTCAG	
**Variant-specific**		
PRSS3-V1 forward	GCCTCGAGATGTGCGGACCTGACGACAG	In this study
PRSS3-V2 forward	GCCTCGAGATGAATCCATTCCTGATCCT	
PRSS3-V3 forward	GCCTCGAGATGCACATGAGAGAGACAAG	
PRSS3-V4 forward	GCCTCGAGATGGGACCTGCGGGGGAGGT	
PRSS3-common reverse	CCTCTAGATTAGCTGTTGGCAGCGATGG	

### Immunofluorescence assay

The colocalization of the EV-A71 3A with PRSS3 was demonstrated byimmunofluorescence assay. The HEK293T cell monolayers were co-transfected with *pLVX::FL-PRSS3-Myc* and *pLVX::FLAG-3A-mCherry*.The cell monolayers co-transfected with *pLVX::FL-PRSS3-Myc* and *pLVX::FLAG-mCherry*served as negative control. At 24 h post-transfection, cells were fixedwith 4% paraformaldehyde, permeabilized with 0.1% Triton X-100, and blocked with 5% bovine serum albumin. Then,the cells were incubated with mouse anti-cMyc antibody followed by Alexa Fluor 488-conjugated secondary antibody. The micrographs were acquired on a Zeiss LSM 800 confocal microscopy (Division of Molecular Medicine, Siriraj, Mahidol University).

### Co-immunoprecipitation assay

The protein–protein interaction of the EV-A71 3A and PRSS3 was confirmed by co-immunoprecipitation assay using anti-Myc-tag monoclonal antibody magnetic beads. Lysates prepared from HEK293T cells separately transfected with the *pLVX-Puro::FL-PRSS3-Myc* and *pLVX-Puro::FLAG-3A-mCherry* in lysis buffer were mixed at 1:1 protein–protein amount ratio. The amounts of protein were normalized by the intensity of reactive bands determined by Western blot analysis and Image Lab Program. The reaction mixtures of lysates containing FL-PRSS3-Myc with either FLAG-mCherry or lysate from untransfected cells served as the controls. The mixtures were incubated with the immune-magnetic beads at 4 °C with constant mixing on a roller shaker for overnight. Unbound fractions were collected. The beads were washed with the wash buffer 1 and 2. Last wash fractions were collected. The lysis and wash buffers supplemented with protease inhibitor cocktails were similar in the pull-down assay. Every magnetic separating step was performed on DynaMag-Spin magnet. The bound proteins were eluted by mixing the beads with SDS-gel reducing loading buffer and boiling for 10 min. The boiled beads were left at room temperature to cool down before spinning down to collect all volume of supernatants containing the co-immunoprecipitated proteins. The co-immunoprecipitated proteins were detected by Western blot analysis using either mouse monoclonal anti-FLAG M2 or anti-cMyc antibody followed by AP-conjugated goat anti-mouse IgG (H + L) antibody. The reactive bands were visualized by adding solution of KPL BCIP/NBT chromogenic substrate.

### Determination of effect of overexpression and siRNA-mediated gene silencing of PRSS3 on EV-A71 replication

Due to the low transfection efficiency in SH-SY5Y cells, HEK293T cells were selected to be used as the model in this experiment for determining the role of neuronal-derived PRSS3 variant 3 in EV-A71 replication.

Prior to performing the infection, HEK293T cells were tested for permissiveness to EV-A71 infection by CCID50 method. RD cells were used as the control of maximum EV-A71 infection. Both HEK293T and RD cells were maintained in complete DMEM and plated at seeding density of 1 × 10^4^ cells/well into wells of 96-well plate. EV-A71 viral stock with titer 10^6.125^ CCID50/100 μL was ten-fold serially diluted from 10^–1^ to 10^–8^ CCID50 determined in HEK293T and RD cells as described previously^59^. The permissiveness of HEK293T cells to EV-A71 infection was determined by comparing CCID50 to those derived from the RD cells.

For overexpression, HEK293T cell monolayers transfected with *pLVX-Puro::FL-PRSS3-Myc* at 5 h post-transfection were adsorbed and infected with EV-A71 MOI 10 for 1 h. Monolayer cells transfected with *pLVX-Puro* vector served as the negative control. Cells treated with transfection reagent alone served as the mock control. At 24 h post-infection, the supernatants were collected for determining virus titer by CCID50 method. Total cellular RNA and protein were extracted from the cells by TRIzol™ reagent following the instruction protocol. The amounts of the viral genome were semi-quantified by one-step SYBR Green-based real-time RT-PCR, qRT-PCR, (Agilent, USA) using the ΔΔCT method to determine fold changes (2^−ΔΔCt^) in copy numbers of EV-A71 genome^60^. The qRT-PCR protocol was as follows; 42 °C for 1 h, 95 °C for 10 min, 40 cycles of 95 °C for 1 min, 57 °C for 45 s, 72 °C for 45 s, and finally subjected to the melting temperature analysis. The sequences of EV-A71 specific primers, namely, EV-F2760 and EV-R3206 were listed in Table 3. Changes in the EV-A71 mRNA expression levels in *pLVX-Puro::FL-PRSS3-Myc*-, *pLVX-Puro*-transfected cells, and mock control cells were calculated after normalization of Ct value to the Ct of internal control and human *GAPDH* house-keeping gene. The normalized EV-A71 Ct (ΔCt) values of the *pLVX-Puro::FL-PRSS3-Myc*-, and *pLVX-Puro*-transfected cells were subtracted with those of mock control cells (ΔΔCt). The fold-changes of viral mRNA (2^-ΔΔCt^) were derived from the subtractive ΔCT of *pLVX-Puro::FL-PRSS3-Myc*-transfected cells relative to those of *pLVX-Puro*-transfected cells.

**Table 3:** Primers used for qRT-PCR.

Primer name	5′ to 3′ sequence	Reference
EV-F2760	ATGGKTATGYWAAYTGGGACAT	^62^
EV-R3206	CCTGACRTGYTTMATCCTCAT	^62^
GAPDH-F	CAAGGTCATCCATGACAACTTTG	In this study
GAPDH-R	GTCCACCACCCTGTTGCTGTA	
Human ACTB-F	GAGCGGGAAATCGTGCGTGACATT	In this study
Human ACTB-R	GAAGGTAGTTTCGTGGATGCC	

For siRNA-mediated gene silencing, HEK293T cell monolayers were transfected with equally pools of three siRNA constructs targeting PRSS3, including Silencer Select pre-designed ID s194719, s11259, and s11260 (Ambion, Life Technologies) at the final concentration 30 nM in the culture medium. Cells incubated with 10 nM siRNA targeting GAPDH house-keeping gene (Silencer Select GAPDH siRNA, Ambion, Life Technologies) served as control for siRNA transfection and irrelevant gene knockdown. The sequences of siRNAs were listed in Table 4. At 72 h post-transfection, total RNA and protein were isolated from the cells using TRIzol reagent following the instruction protocol. The gene knockdown efficiency was determined by qRT-PCR using the ΔΔCT method to determine fold changes (2^-ΔΔCt^) of *PRSS3* and *GAPDH* expressions. The fold-changes of *PRSS3* and *GAPDH* expressions were the Ct values of those two genes of siRNA-treated samples normalized with the internal ß-actin house-keeping gene (*ACTB*) relative to the normalized Ct of the mock control. The sequences of the specific primers were listed in Table 3. The expression of GAPDH and PRSS3 proteins was determined by Western blot analysis using ECL detection system. The intensity of GAPDH , PRSS3, and ß-actin reactive bands were determined by Image Lab Program. The PRSS3 and GAPDH bands were normalized with the respective ß-actin and calculated the fold-change relative to mock. At 48 h post-transfection, the siRNA-transfected cells and mock control were infected with EV-A71 at MOI 10. The siRNA-transfected cells and the mock control uninoculated with the virus served as the negative infection control. At 24 h post-infection, the supernatants were collected for measuring virus titer by CCID50 method. The cells were treated with TRIzol reagent to extract total RNA and proteins. The RNA samples were semi-quantified for EV-A71 mRNA by qRT-PCR using 2^−ΔΔCt^.

**Table 4:** Sequences of siRNA against *PRSS3* (Ambion, Life Technologies).

siRNA ID	Strand	5′ to 3′ sequence
s194719	Sense	AGAUCAUCCGCCACCCUAAtt
	Anti-sense	UUAGGGUGGCGGAUGAUCUtg
s11259	Sense	CUCUGAGCUUUGGUGCUGAtt
	Anti-sense	UCAGCACCAAAGCUCAGAGtg
s11260	Sense	GGGAGAGCACAACAUCAAAtt
	Anti-sense	UUUGAUGUUGUGCUCUCCCag

The protein samples derived from the overexpression and siRNA-mediated gene silencing experiments were subjected to Western blot analysis to measure the levels of viral capsid protein VP0 and its cleaved form, VP2. The blotted membranes were probed with either mouse anti-EV-A71 VP2 and anti-ß-actin antibody. The intensity of VP2 and ß-actin reactive bands were determined by Image Lab Program. The fold-changes of EV-A71 VP2 were determined relative to ß-actin protein. All qRT-PCR data were derived from technical triplicates of each sample conducted by three independent experiments and expressed in mean ± SD.

The viability and proliferation of the HEK293T cells that overexpressed PRSS3 and *PRSS3*-knocked down as well as controls at 24 h post-transfection was measured by sulforhodamine B (SRB) assay as described previously^32^.

## Supplementary Information

Supplementary Information 1.

Supplementary Information 2.

Supplementary Information 3.

Supplementary Information 4.

Supplementary Information 5.

Supplementary Information 6.

## Data Availability

Data generated or analyzed in this study are included in this published article both in text and Supplementary Information files as mentioned in the main text. The DNA sequencing data of *PRSS3 variant 3* and *EV-A71 3A* nucleotide sequences were deposited in the GenBank repository under the accession numbers ON227450 and ON227451, respectively.
