# Supplementary material for: Host neuronal PRSS3 interacts with enterovirus A71 3A protein and its role in viral replication
Source: Sci Rep. 2022 Jul 27;12:12846. doi: 10.1038/s41598-022-17272-2 (PMC9328647; doi:10.1038/s41598-022-17272-2)
Supplement: Supplementary file 1 — Supplementary Information 1. [file 41598_2022_17272_MOESM1_ESM.pdf]

Host neuronal PRSS3 interacts with enterovirus A71 3A protein and its role in viral replication

Patthaya Rattanakomol[1], Potjanee Srimanote[1,2], Pongsri Tongtawe[1], Onruedee Khantisitthiporn[2,3], Oratai Supasorn[1] & Jeeraphong Thanongsaksrikul[1,2*]

[1]Graduate Program in Biomedical Sciences, Faculty of Allied Health Sciences, Thammasat University, Pathum Thani, 12120, Thailand
[2]Thammasat University Research Unit in Molecular Pathogenesis and Immunology of Infectious Diseases, Thammasat University, Pathum Thani, 12120, Thailand
[3]Department of Medical Technology, Faculty of Allied Health Sciences, Thammasat University, Pathum Thani, 12120, Thailand

*** Correspondence:** JeeraphongThanongsaksrikul
jeeraphong.t@allied.tu.ac.th

Supplementary Data S1 Prediction of trypsin cleavage sites on EV-A71 polyprotein by PeptideCutter.

Polyprotein [Enterovirus A71] GenBank Accession number: AEQ59479.1

Amino acid position of each viral mature protein

1-69	=	VP4
70-323	=	VP2
324-565	=	VP3
566-862	=	VP1
863-1012	=	2A
1013-1111	=	2B
1112-1440	=	2C
1441-1526	=	3A
1527-1548	=	3B
1549-1731	=	3C
1732-2193	=	3D

There were 194 positions in amino acid sequence of EV-A71 polyprotein that were predicted as trypsin cleavage sites. The predicted positions, amino acid sequences, lengths and masses of the cleaved peptides were listed in the table.

Position of cleavage site	Resulting peptide sequence	Peptide length [aa]	Peptide mass [Da]
9	MGSQVSTQR	9	993.103
34	SGSHENSNSATEGSTINYTTINYYK	25	2738.817
43	DSYAATAGK	9	882.926
47	QSLK	4	474.558
52	QDPDK	5	601.614
58	FANPVK	6	674.798

69	DIFTEMAAPLK	11	1235.461
81	SPSAEACGYSDR	12	1242.282
131	VAQLTIGNSTITTQEA<br>ANIIVGYGEWPSYCSD<br>DDATAVDKPTRPDVSV<br>NR	50	5340.818
138	FYTLDTK	7	887.000
142	LWEK	4	574.677

Position of cleavage site	Resulting peptide sequence	Peptide length [aa]	Peptide mass [Da]
145	SSK	3	320.346
150	GWYWK	5	738.844
172	FPDVLTETGVFGQNAQ FHYLYR	22	2602.888
185	SGFCIHVQCNASK	13	1393.598
218	FHQGALLVAILPEYVI GTVAGGTGTEDSHPPY K	33	3438.885
255	QTQPGADGFELQHPYV LDAGIPISQLTICPHQ WINLR	37	4157.716
318	TNNCATIIVPYMNTLP FDSALNHCNFGLLVVP ISPLDFDQGATPVIPI TITLAPMCSEF AGLR	63	6749.893
364	QAVTQGFPTEPKPGTN QFLTTDDGVSAPILPN FHPTPCIHIPGEVR	46	4926.538
391	NLLELCQVETILEVNN VPTNATSLMER	27	3044.485
393	IR	2	287.362
402	FPVSAQAGK	9	904.034
410	GELCAVFR	8	894.056
415	ADPGR	5	514.539
453	NGPWQSTLLGQLCGYY TQWSGSLEVTFMFTGS FMATGK	38	4195.748
467	MLIAYTPPGGPLPK	14	1454.791
469	DR	2	289.291

Position of cleavage site	Resulting peptide sequence	Peptide length [aa]	Peptide mass [Da]
501	ATAMLGTHVIWDFGLQSSVTLVIPWISNTHYR	32	3615.167
505	AHAR	4	453.501
545	DGVFDYYTTGLVSIWYQTNYVVPIGAPNTAYIIALAAAQK	40	4368.953
550	NFTMK	5	639.767
568	LCKDTSHILQTASIQGDR	18	1986.230
583	VADVIESSIGDSVSR	15	1533.656
587	ALTR	4	459.546
603	ALPAPTGQNTQVSSHR	16	1663.811
632	LDTGEVPALQAAEIGASSNTSDESMIETR	29	2993.203
651	CVLNSHSTAETTLDSFFSR	19	2115.302
685	AGLVGEIDLPLEGTTNPNGYANWDIDITGYAQMR	34	3666.033
686	R	1	174.203
687	K	1	146.189
695	VELFTYMR	8	1058.261
731	FDAEFTFVACTPTGEVVPQLLQYMFVPPGAPKPDSR	36	3956.543
747	ESLAWQTATNPSVFVK	16	1777.995
780	LTDPPAQVSVPFMSPASAYQWFYDGYPTFGEHK	33	3735.141

Position of cleavage site	Resulting peptide sequence	Peptide length [aa]	Peptide mass [Da]
783	QEK	3	403.436
801	DLEYGACPNNMMGTFS VR	18	2005.266
807	TVGSSK	6	577.635
809	SK	2	233.268
815	YPLVVR	6	745.920
819	IYMR	4	581.731
821	MK	2	277.382
824	HVR	3	410.476
832	AWIPRPMR	8	1026.268
839	NQNYLFK	7	926.040
856	ANPNYAGNSIKPTGTS R	17	1747.885
864	TAITTLGK	8	803.954
878	FGQQSGAIYVGNFR	14	1543.702
882	VVNR	4	486.572
900	HLATHNDWANLVWEDS SR	18	2151.282
917	DLLVSSTTAQGCDTIA R	17	1750.941
930	CDCQTGVYYCNSK	13	1483.649
931	R	1	174.203
932	K	1	146.189
955	HYPVSFSKPSLIYVEA SEYYPAR	23	2704.034

Position of cleavage site	Resulting peptide sequence	Peptide length [aa]	Peptide mass [Da]
977	YQSHLMLAAGHSEPGD CGGILR	22	2312.603
1000	CQHGVVGIVSTGGNGL VGFADVR	23	2242.538
1019	DLLWLDEEAMEQGVSD YIK	19	2254.493
1035	GLGDAFGTGFTDAVSR	16	1570.679
1041	EVEALR	6	715.805
1053	NHLIGSDGAVEK	12	1239.351
1056	ILK	3	372.508
1060	NLIK	4	486.612
1070	LISALVIVIR	10	1096.422
1096	SDYDMVTLTATLALIG CHGSPWAWIK	26	2850.300
1098	AK	2	217.268
1110	TASILGIPIAQK	12	1211.467
1117	QSASWLK	7	818.928
1118	K	1	146.189
1127	FNDMASAAK	9	954.066
1135	GLEWISNK	8	946.071
1138	ISK	3	346.427
1144	FIDWLR	6	849.000
1146	EK	2	275.305
1152	IVPAAR	6	625.769
1154	EK	2	275.305
1162	AEFLTNLK	8	935.088

Position of cleavage site	Resulting peptide sequence	Peptide length [aa]	Peptide mass [Da]
1200	QLPLLENQITNLEQSAASQEDLEAMFGNVSYLAHFCRK	38	4309.835
1210	FQPLYATEAK	10	1167.327
1211	R	1	174.203
1217	VYVLEK	6	749.905
1218	R	1	174.203
1226	MNNYMQFK	8	1075.265
1228	SK	2	233.268
1230	HR	2	311.344
1239	IEPVCLIIR	9	1055.344
1246	GSPGTGK	7	602.645
1255	SLATGIIAR	9	901.074
1260	AIADK	5	516.595
1279	YHSSVYSLPPDPDHFDGYK	19	2224.371
1296	QQVVTVMDDLCQNPDGK	17	1890.113
1320	DMSLFCQMVSTVDFIPPMASLEEK	24	2719.193
1327	GVSFTSK	7	724.812
1350	FVIASTNSSNIIVPTVSDSDAIR	23	2406.675
1352	RR	2	330.390
1366	FYMDCDIEVTDSYK	14	1728.906
1371	TDLGR	5	560.608
1376	LDAGR	5	530.581

Position of cleavage site	Resulting peptide sequence	Peptide length [aa]	Peptide mass [Da]
1379	AAK	3	288.347
1390	LCSENNTANFK	11	1240.353
1391	R	1	174.203
1399	CSPLVCGK	8	806.006
1404	AIQLR	5	599.731
1406	DR	2	289.291
1407	K	1	146.189
1409	SK	2	233.268
1411	VR	2	273.335
1423	YSVDTVVSELIR	12	1380.561
1428	EYNSR	5	667.676
1444	SAIGNTIEALFQGPPK	16	1642.872
1449	FRPIR	5	687.843
1474	ISLEEKPAPDAISDLL ASVDSEEVR	25	2683.949
1478	QYCR	4	568.648
1493	EQGWIIPETPINVER	15	1780.998
1497	HLNR	4	538.607
1520	AVLVMQSIATVVAVVS LVYVIYK	23	2466.061
1534	LFAGFQGAYSGAPK	14	1413.596
1538	QVLR	4	514.625
1543	KPVLR	5	611.786
1560	TATVQGPSLDFALSLL R	17	1789.062

Position of cleavage site	Resulting peptide sequence	Peptide length [aa]	Peptide mass [Da]
1561	R	1	174.203
1564	NIR	3	401.466
1579	QVQTDQGHFTMLGVR	15	1716.932
1587	DHLAVLPR	8	920.079
1593	HAQPGK	6	636.709
1600	TIWVEHK	7	912.056
1630	LVNVLDAVELVDEQGV NLELTLVTLDTNEK	30	3296.717
1632	FR	2	321.379
1636	DITK	4	475.542
1682	FIPETISGASDATLVI NTEHMPSMFVPVGDVV QYGFLNLSGKPTHR	46	4974.681
1691	TMMYNFPTK	9	1132.358
1704	AGQCGGVVTSVGK	13	1162.326
1715	IVGIHIGGNGR	11	1092.266
1723	QGFCAGLK	8	822.977
1724	R	1	174.203
1738	SYFASVQGEIQWVK	14	1641.844
1741	SNK	3	347.371
1745	ETGR	4	461.475
1753	LNINGPTR	8	884.003
1755	TK	2	247.294
1766	LEPSVFHDVFK	11	1317.507
1769	GSK	3	290.319

Position of cleavage site	Resulting peptide sequence	Peptide length [aa]	Peptide mass [Da]
1777	EPAVLTSK	8	843.976
1780	DPR	3	386.408
1792	LEVDFEQALFSK	12	1425.601
1817	YVGNVLHEPDEYVTQA ALHYANQLK	25	2873.174
1825	QLDINTSK	8	918.015
1857	MSMEEACYGTENLEAI DLCTSAGYPYSALGIK	32	3431.864
1858	K	1	146.189
1859	R	1	174.203
1867	DILDPVTR	8	928.053
1871	DVSK	4	447.489
1873	MK	2	277.382
1878	FYMDK	5	702.823
1890	YGLDLPYSTYVK	12	1418.609
1898	DELRPLDK	8	985.105
1900	IK	2	259.349
1901	K	1	146.189
1903	GK	2	203.241
1905	SR	2	261.281
1919	LIEASSLNDSVYLR	14	1579.770
1951	MTFGHLYEVFHANPGT VTGSAVGCNPDVFWSK	32	3469.894
1978	LPILLPGSLFAFDYSG YDASLSPVWFR	27	3032.488

Position of cleavage site	Resulting peptide sequence	Peptide length [aa]	Peptide mass [Da]
1985	ALEVVLR	7	798.981
2008	EIGYTEEAVSLIEGIN HTHHVYR	23	2667.918
2010	NK	2	260.293
2038	TYCVLGGMPSGCSGTS IFNSMINNIIIR	28	2949.466
2043	TLLIK	5	586.773
2046	TFK	3	394.471
2077	GIDLDELNMVAYGDDV LASYPFPIDCLELAK	31	3400.860
2080	TGK	3	304.346
2091	EYGLTMTPADK	11	1225.379
2107	SPCFNEVTWENATFLK	16	1886.109
2108	R	1	174.203
2127	GFLPDHQFPFLIHPTM PMR	19	2281.721
2134	EIHESIR	7	882.972
2137	WTK	3	433.508
2140	DAR	3	360.370
2147	NTQDHVR	7	868.905
2158	SLCLLAWHNGK	11	1241.473
2163	DEYEK	5	682.685
2169	FVSTIR	6	721.855
2175	SVPVGK	6	585.701
2186	ALAIPSFENLR	11	1230.429
2187	R	1	174.203
	NWLELF	6	820.943
