# Supplementary material for: Host neuronal PRSS3 interacts with enterovirus A71 3A protein and its role in viral replication
Source: Sci Rep. 2022 Jul 27;12:12846. doi: 10.1038/s41598-022-17272-2 (PMC9328647; doi:10.1038/s41598-022-17272-2)
Supplement: Supplementary file 2 — Supplementary Information 2. [file 41598_2022_17272_MOESM2_ESM.pdf]

# Host neuronal PRSS3 interacts with enterovirus A71 3A protein and its role in viral replication

**Patthaya Rattanakomol[1], Potjanee Srimanote[1,2], Pongsri Tongtawe[1], Onruedee Khantisitthiporn[2,3], Oratai Supasorn[1] & Jeeraphong Thanongsaksrikul[1,2*]**

[1]Graduate Program in Biomedical Sciences, Faculty of Allied Health Sciences, Thammasat University, Pathum Thani, 12120, Thailand
[2]Thammasat University Research Unit in Molecular Pathogenesis and Immunology of Infectious Diseases, Thammasat University, Pathum Thani, 12120, Thailand
[3]Department of Medical Technology, Faculty of Allied Health Sciences, Thammasat University, Pathum Thani, 12120, Thailand

*** Correspondence:** JeeraphongThanongsaksrikul
jeeraphong.t@allied.tu.ac.th

**Supplementary Data S2** Prediction of EV-A71 2A$^{pro}$ and 3C$^{pro}$ cleavage sites on human trypsinogen PRSS3 variant 3 (PRSS3-V3).

Amino acid sequence of PRSS3-V3 (261 aa)

```
1   MHMRETSGFT LKKGRSAPLV FHPPDALIAV PFDDDDKIVG
41  GYTCEENSLP YQVSLNSGSH FCGGSLISEQ WVVSAAHCYK
81  TRIQVRLGEH NIKVLEGNEQ FINAAKIIRH PKYNRDTLDN
121 DIMLIKLSSP AVINARVSTI SLPTAPPAAG TECLISGWGN
161 TLSFGADYPD ELKCLDAPVL TQAECKASYP GKITNSMFCV
201 GFLEGGKDSC QRDSGGPVVC NGQLQGVVSW GHGCAWKNRP
241 GVYTKVYNYV DWIKDTIAAN S
```

Structurally, each variant of human PRSS3 differs only at N-terminal leader sequence. The major features of trypsinogens are a negatively charged activation peptide with a typical enterokinase cleavage site (DDDDKI↓I) and three conserved catalytic triad residues (His, Asp, Ser). Arginine (R) at position 212 of PRSS3 variant 3., comparable to the position 198 of the reported PRSS3 variant 2 (NCBI Reference Sequence: NM_002771.4), is a unique residue in human PRSS3 which is responsible for inhibitor resistance (DOI 10.1007/s00018-007-7288-3, DOI:https://doi.org/10.1074/jbc.272.16.10573).

EV-A71 2A$^{pro}$ and 3C$^{pro}$ proteins are chymotrypsin-like proteases. The consensus motifs for enzymatic cleavage of the proteins are defined as P1 and P1' position. For EV-A71 2A$^{pro}$, the preferential amino acid residues at P1 are threonine (T), tyrosine (Y), and phenylalanine (F) while amino acid at P1' is strict for glycine (G). For EV-A71 3C$^{pro}$, P1 position is specific for glutamine (Q) while P1' is for glycine (G) > alanine (A) > serine (S) (https://doi.org/10.1371/journal.ppat.1008927). Evaluation of cleavage sites of the EV-A71 proteases on PRSS3-V3 found that there are 1 cleavage site of 2A$^{pro}$ at amino acid position 164 (F-G) and 2 cleavage sites of 3C$^{pro}$ at position 182 (Q-A) and 225 (Q-G).
