# Supplementary material for: Host neuronal PRSS3 interacts with enterovirus A71 3A protein and its role in viral replication
Source: Sci Rep. 2022 Jul 27;12:12846. doi: 10.1038/s41598-022-17272-2 (PMC9328647; doi:10.1038/s41598-022-17272-2)
Supplement: Supplementary file 3 — Supplementary Information 3. [file 41598_2022_17272_MOESM3_ESM.pdf]

# Host neuronal PRSS3 interacts with enterovirus A71 3A protein and its role in viral replication

**Patthaya Rattanakomol[1], Potjanee Srimanote[1,2], Pongsri Tongtawe[1], Onruedee Khantisitthiporn[2,3], Oratai Supasorn[1] & Jeeraphong Thanongsaksrikul[1,2*]**

[1]Graduate Program in Biomedical Sciences, Faculty of Allied Health Sciences, Thammasat University, Pathum Thani, 12120, Thailand

[2]Thammasat University Research Unit in Molecular Pathogenesis and Immunology of Infectious Diseases, Thammasat University, Pathum Thani, 12120, Thailand

[3]Department of Medical Technology, Faculty of Allied Health Sciences, Thammasat University, Pathum Thani, 12120, Thailand

*** Correspondence:** JeeraphongThanongsaksrikul
jeeraphong.t@allied.tu.ac.th

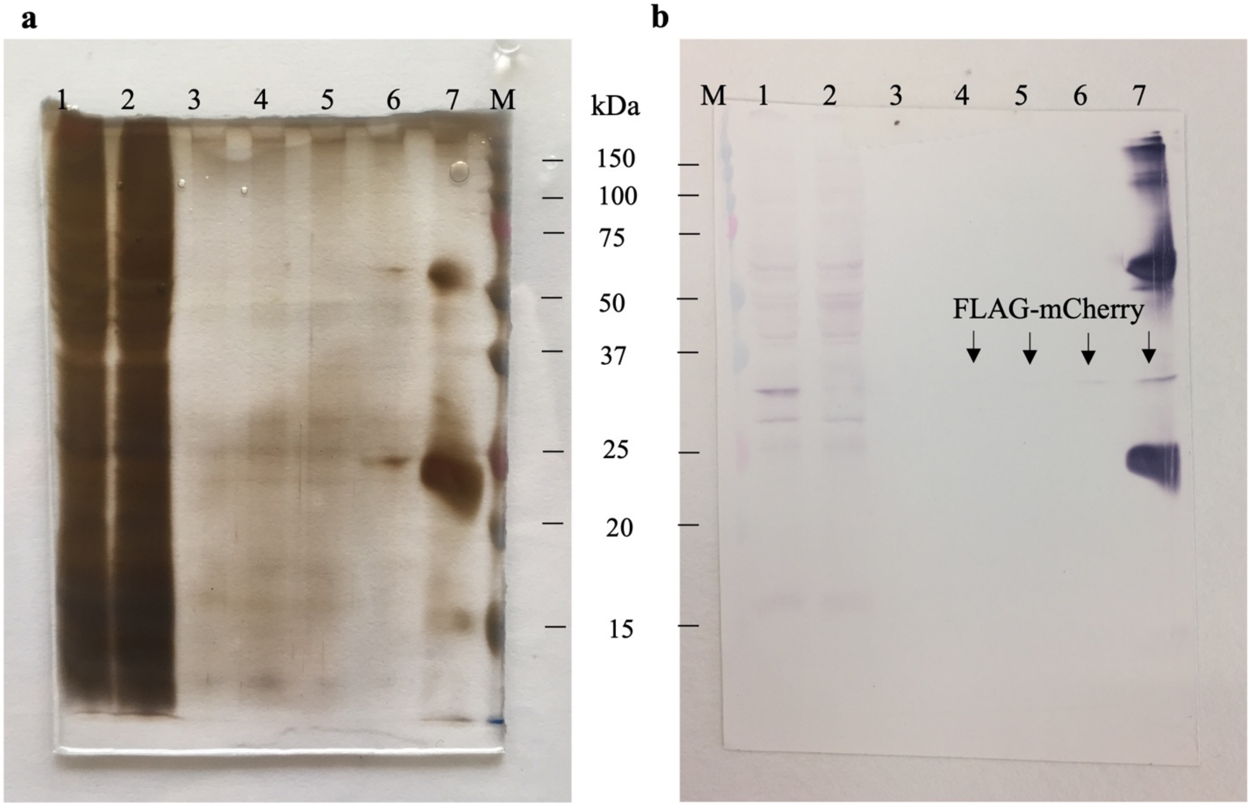


**Supplementary Fig. 1.** Pull down of proteins with FLAG-mCherry in the cell lysate prepared from *pLVX-Puro::FLAG-mCherry*-transfected SH-SY5Y cells. The protein fractions were resolved in 12% SDS-PAGE as replica wells on the same gel separated with standard protein marker (lane M). The two half gel was cut at the lane M. (**a**) the first half of the full-length gel was stained with Coomassie brilliant blue followed by counterstaining with silver dye. (**b**) the respective protein fractions on the other half of the gel were transferred into nitrocellulose membrane. The blotted membrane was incubated with rabbit anti-mCherry polyclonal antibody to detect the FLAG-mCherry (indicated by arrows). Lanes 1, cell lysate prepared from *pLVX-Puro::FLAG-mCherry* transfected SH-SY5Y cells; lanes 2, flow-through fraction; lanes 4-6, eluate fractions from pull down by anti-FLAG M2 magnetic beads; lanes 3, last washed fraction; lanes 7, the last eluate fraction form boiled anti-FLAG M2 magnetic beads.
