# Supplementary material for: Host neuronal PRSS3 interacts with enterovirus A71 3A protein and its role in viral replication
Source: Sci Rep. 2022 Jul 27;12:12846. doi: 10.1038/s41598-022-17272-2 (PMC9328647; doi:10.1038/s41598-022-17272-2)
Supplement: Supplementary file 4 — Supplementary Information 4. [file 41598_2022_17272_MOESM4_ESM.pdf]

Host neuronal PRSS3 interacts with enterovirus A71 3A protein and its role in viral replication

Patthaya Rattanakomol[1], Potjanee Srimanote[1,2], Pongsri Tongtawe[1], Onruedee Khantisitthiporn[2,3], Oratai Supasorn[1] & Jeeraphong Thanongsaksrikul[1,2*]

[1]Graduate Program in Biomedical Sciences, Faculty of Allied Health Sciences, Thammasat University, Pathum Thani, 12120, Thailand

[2]Thammasat University Research Unit in Molecular Pathogenesis and Immunology of Infectious Diseases, Thammasat University, Pathum Thani, 12120, Thailand

[3]Department of Medical Technology, Faculty of Allied Health Sciences, Thammasat University, Pathum Thani, 12120, Thailand

*** Correspondence:** JeeraphongThanongsaksrikul
jeeraphong.t@allied.tu.ac.th

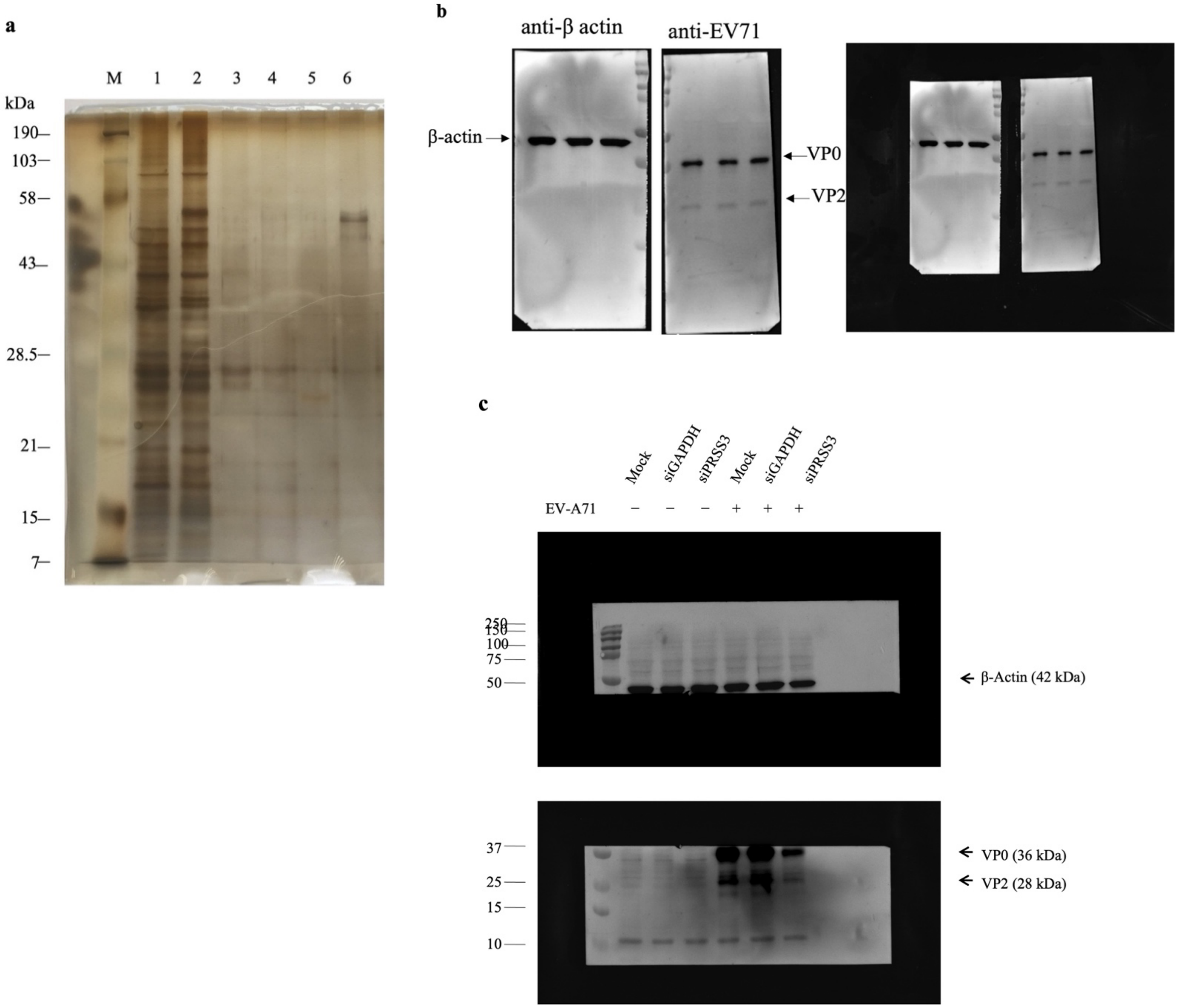

Supplementary Fig. 2. Original images of (**a**) Fig. 1a, (**b**) Fig. 6c, and (**c**) Fig. 7d. Panels (**b**) and (**c**) were the images of blot detected by enhanced chemiluminescence system were documented by ChemiDoc MP (Bio-Rad). The images with adequate length of Fig. 7d were absent because the blotted membranes were cut prior to hybridization with antibodies.
