# Supplementary material for: Host neuronal PRSS3 interacts with enterovirus A71 3A protein and its role in viral replication
Source: Sci Rep. 2022 Jul 27;12:12846. doi: 10.1038/s41598-022-17272-2 (PMC9328647; doi:10.1038/s41598-022-17272-2)
Supplement: Supplementary file 6 — Supplementary Information 6. [file 41598_2022_17272_MOESM6_ESM.pdf]

Host neuronal PRSS3 interacts with enterovirus A71 3A protein and its role in viral replication

Patthaya Rattanakomol[1], Potjanee Srimanote[1,2], Pongsri Tongtawe[1], Onruedee Khantisitthiporn[2,3], Oratai Supasorn[1] & Jeeraphong Thanongsaksrikul[1,2*]

[1]Graduate Program in Biomedical Sciences, Faculty of Allied Health Sciences, Thammasat University, Pathum Thani, 12120, Thailand

[2]Thammasat University Research Unit in Molecular Pathogenesis and Immunology of Infectious Diseases, Thammasat University, Pathum Thani, 12120, Thailand

[3]Department of Medical Technology, Faculty of Allied Health Sciences, Thammasat University, Pathum Thani, 12120, Thailand

*** Correspondence:** JeeraphongThanongsaksrikul
jeeraphong.t@allied.tu.ac.th

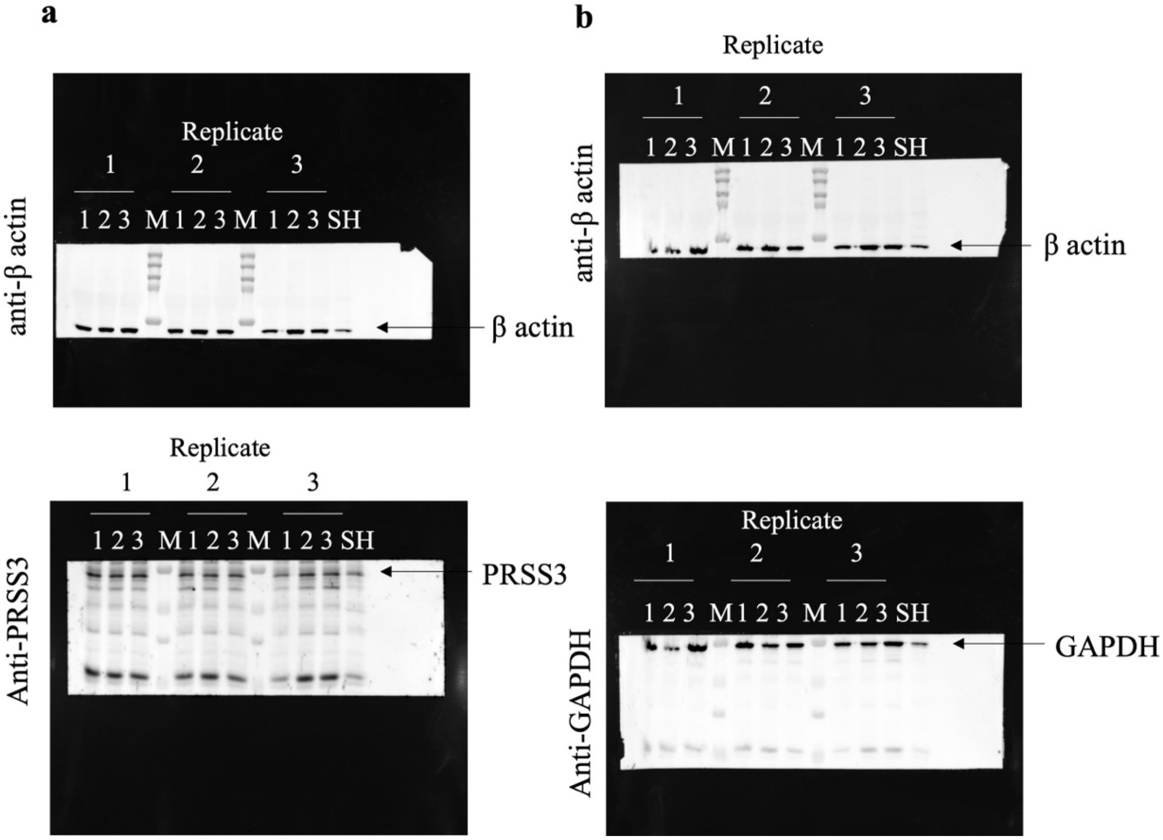

Supplementary Fig. 4. Total proteins extracted from *siPRSS3*-treated- and *siGAPDH*-treated cells and mock control cells were determined for protein expression of PRSS3 (**a**) and GAPDH (**b**) by Western blot analysis. Protein bands of the ß-actin of the respective samples were used for normalization. The intensity of GAPDH , PRSS3, and ß-actin reactive bands were determined by Image Lab Program. The intensity of PRSS3 and GAPDH bands was normalized with the respective ß-actin and calculated the fold-change relative to mock. The images with adequate length were absent because the blotted membranes were cut prior to hybridization with antibodies. The images were captured and taken from ChemiDoc MP (Bio-Rad). The data were form three replicate experiments. Lanes 1; protein samples from mock, lanes 2; protein samples from *siGAPDH*-treated cells, lanes3; protein samples from *siPRSS3*-treated cells, lanes M; protein standard marker, and lanes SH; protein samples from normal SH-SY5Ycells served as control.
